# *Proteus* sp. Strain JHY1 Synergizes with Exogenous Dopamine to Enhance Rice Growth Performance Under Salt Stress

**DOI:** 10.3390/microorganisms13081820

**Published:** 2025-08-04

**Authors:** Jing Ji, Baoying Ma, Runzhong Wang, Tiange Li

**Affiliations:** 1 School of Environmental Science and Engineering, Tianjin University, Tianjin 300072, China; baoyingma@tju.edu.cn (B.M.); 2022214002@tju.edu.cn (R.W.); 2022214062@tju.edu.cn (T.L.); 2 Haihe Laboratory of Sustainable Chemical Transformations, Tianjin 300192, China; 3 Frontiers Science Center for Synthetic Biology, Tianjin University, Tianjin 300072, China

**Keywords:** PGPR, dopamine, salt stress, salt-tolerant strain, rice, photosynthesis, antioxidant defense, osmotic regulation, rhizosphere environment

## Abstract

Soil salinization severely restricts crop growth and presents a major challenge to global agriculture. In this study, a plant-growth-promoting rhizobacterium (PGPR) was isolated and identified as *Proteus* sp. through 16S rDNA analysis and was subsequently named *Proteus* sp. JHY1. Under salt stress, exogenous dopamine (DA) significantly enhanced the production of indole-3-acetic acid and ammonia by strain JHY1. Pot experiments revealed that both DA and JHY1 treatments effectively alleviated the adverse effects of 225 mM NaCl on rice, promoting biomass, plant height, and root length. More importantly, the combined application of DA-JHY1 showed a significant synergistic effect in mitigating salt stress. The treatment increased the chlorophyll content, net photosynthetic rate, osmotic regulators (proline, soluble sugars, and protein), and reduced lipid peroxidation. The treatment also increased soil nutrients (ammoniacal nitrogen and available phosphorus), enhanced soil enzyme activities (sucrase and alkaline phosphatase), stabilized the ion balance (K^+^/Na^+^), and modulated the soil rhizosphere microbial community by increasing beneficial bacteria, such as Actinobacteria and Firmicutes. This study provides the first evidence that the synergistic effect of DA and PGPR contributes to enhanced salt tolerance in rice, offering a novel strategy for alleviating the adverse effects of salt stress on plant growth.

## 1. Introduction

The issue of soil salinization is becoming increasingly prevalent in the context of climate change and global population growth. Approximately 20% of global agricultural land has been affected by salinization, which is projected to rise to 50% by 2050 [1,2]. This issue is particularly pressing in regions where rice is a major crop, as salinization not only threatens agricultural sustainability but also poses a significant challenge to global food security. The phenomenon of salinization has resulted in considerable losses in agricultural production, with annual economic losses estimated at USD 27.3 billion [3]. The accumulation of salts in the soil not only alters the physical and chemical properties of the soil, reducing soil aeration and water retention capacity, but also adversely affects soil fertility and microbial diversity [2,4]. Consequently, the accumulation of salts impairs the soil’s ability to supply water and nutrients to plants, ultimately hindering plant growth and development. In addition, it has been found that high concentrations of salt could inhibit cell division and elongation, significantly hindering the development of plant roots [5]. Furthermore, previous studies have found that under salt stress, the protein synthesis pathway in wheat was inhibited. At the same time, the expression of ubiquitin-activating enzyme E1 is significantly increased, resulting in enhanced protein degradation and a substantial reduction in protein content [6]. Salt stress could also disrupt the normal metabolic processes of plants, such as reducing the activity of ribulose-1,5-bisphosphate carboxylase, thereby inhibiting photosynthesis, which in turn reduces organic matter accumulation and ultimately leads to a decrease in biomass [7]. Therefore, addressing the adverse effects of soil salinity on food crops is not only essential for maintaining agricultural productivity but also a critical step toward ensuring global food security in the face of climate change and population growth.

Plant-growth-promoting rhizobacteria (PGPR) serve as natural soil microbial engineers, significantly contributing to the enhancement of plant growth and development. Additionally, PGPR enhance plant tolerance to various environmental stresses, including salinity, drought, and heavy metal stress [8,9]. As a result, PGPR emerge as an environmentally friendly biofertilizer, which could be used to replace chemical fertilizers. If PGPR can be applied to agricultural cultivation, it will provide a more sustainable and environmentally friendly solution for crop cultivation. In recent years, several studies have demonstrated that PGPR could reduce ethylene levels within plants by producing 1-aminocyclopropane-1-carboxylate (ACC) deaminase, which converts the stress signaling molecule ACC into α-ketobutyrate and ammonia. PGPR also contribute to the synthesis of indole-3-acetic acid (IAA), which promotes root development and plant growth. Moreover, these beneficial bacteria could enhance nutrient uptake in plants by nitrogen fixation, phosphate solubilization, and siderophore secretion, thereby promoting plant growth and yield [10,11,12]. These studies indicated that PGPR had broad application prospects in enhancing the stress resistance of crops. In addition, PGPR, such as *Bacillus amyloliquefaciens* and *Bacillus licheniformis*, was capable of producing extracellular polysaccharides (EPSs). These EPSs formed biofilms in the maize rhizosphere, enhancing various physiological traits, including photosynthetic efficiency and antioxidant enzyme activity, thereby improving maize tolerance to drought and salinity [13]. However, complex growing environments and salt stress could impose limitations on the growth of PGPR, so strategies to mitigate these limitations need to be explored [14].

While traditional research has long established dopamine (DA) as a crucial substance in the nervous systems of humans and animals [15], recent studies have also revealed its significant role in plants. Existing studies have shown that the application of DA could increase superoxide dismutase (SOD) activity in soybean roots, thereby reducing reactive oxygen species (ROS) levels and lipid peroxidation levels. Another study demonstrated that the application of DA to tomato seedlings under salt stress could significantly enhance leaf relative water content (RWC), soil and plant analyzer development, chlorophyll-a, chlorophyll-b, and total chlorophyll content. Furthermore, DA treatment could increase the endogenous IAA concentration while reducing the abscisic acid level in tomato plants [16]. Previous research suggested that exogenously applied DA could also enhance salt tolerance in plants. It increased the RWC in rice by reducing the expression of the water channel protein *OsPIP1-3*, thereby improving the salt tolerance of rice [17]. Additionally, research has shown that priming rice seeds under salt stress with DA could effectively promote seed germination and seedling growth [18]. Previous research indicated that DA could also improve the salt tolerance in soybean by regulating molecular expression [19]. Moreover, DA could promote the colonization of arbuscular mycorrhizal fungi on apple roots and improve the salt tolerance of apples [20,21]. Notably, DA could recruit beneficial soil microorganisms and modulate soil microbial communities [22,23,24]. These findings revealed the dual potential of DA: it not only served as a regulator of plant physiology but also emerged as a potential factor in promoting rhizosphere microbial activity. This discovery holds significant implications for enhancing crop stress resistance and advancing the development of sustainable agriculture.

Rice (*Oryza sativa*) is one of the world’s most important staple crops, serving as the primary food source for more than half of the world’s population [25]. However, most rice-growing regions, including Southeast Asia, South Asia, and China, are increasingly challenged by severe salinity stress [26,27,28]. Given the limitations of current chemical-based salinity mitigation strategies and their potential environmental risks, the development of biological strategies, such as PGPR and bioactive compounds like DA, is crucial for achieving sustainable crop production in saline soils. However, research on the effects of DA under salt stress in rice remains very limited, and to date, no studies have investigated the combined application of DA and PGPR in alleviating salt stress in rice. Considering DA’s potent antioxidant properties in plants and the potential for synergistic interactions with PGPR, this study, for the first time, explored the enhancing effects of exogenous DA in combination with salt-tolerant PGPR strains on the growth performance of rice under salt stress, aiming to provide a novel and eco-friendly strategy for improving crop resilience in saline environments. This study aims to (1) screen for PGPR with excellent growth-promoting traits and assess their growth characteristics and plant-growth-promoting (PGP) characteristics; (2) analyze the effects of different salinity levels with/without DA on the growth status and plant-growth-promoting indicators of PGPR; (3) evaluate the individual effects of DA application and PGPR inoculation on plant growth, morphological parameters, oxidative stress levels, osmotic substances accumulation, soil nutrient status, Na^+^ and K^+^ concentrations in plants and soil, rhizosphere soil enzymatic activities, and rhizosphere microbial community; and (4) investigate the potential physiological mechanism by which the combined effect of DA and PGPR improved the salt tolerance of rice by analyzing various physiological indicators.

## 2. Materials and Methods

### 2.1. Isolation and Identification of Salt-Tolerant PGPR

In order to screen for salt-tolerant PGPR, fresh rhizosphere soil samples were collected from the experimental field at Tianjin University using sterile bags. The samples were obtained from the rhizosphere of *Iris tectorum* Maxim. (Iris), an important salt-tolerant species. A 10 g sample of the soil was taken and added to 200 mL of sterile water in a conical flask, while the remaining soil samples were stored at 4 °C. The conical flask was placed on a magnetic stirrer and stirred for 20 min, and then left to stand still for 40 min. After that, the liquid was collected and centrifuged at 4000× *g* for 10 min at 4 °C. The supernatant was then collected and serially diluted to 10^−5^ under sterile conditions. Subsequently, 1 mL of the diluted solution was added to 100 mL of Luria–Bertani (LB) medium with NaCl concentrations of 50, 150, 250, and 350 mM. The bacterial culture shake flasks were incubated at 28 °C and 180 rpm for 48 h. After incubation, 50 µL of the culture was spread on LB agar plates. To obtain a single colony of bacteria, individual colonies from the plate were picked and subjected to three rounds of streak plating. The purified strains were then inoculated into Dworkin and Foster medium supplemented with 3 mM ACC instead of (NH_4_)_2_SO_4_ (ADF medium) and cultured at 28 °C and 180 rpm for 24 h to obtain the strains which exhibited high ACC deaminase activity. A total of five strains were screened, among which strain JHY1 demonstrated the optimal growth performance and was consequently chosen for the follow-up research. The strain JHY1 was subsequently cultured in LB broth to OD 0.6–0.8, then diluted to 10^−6^ and plated on LB agar plates to observe colony morphology. Gram staining was performed using the Biosharp 125 Gram staining kit (catalog no. BL734A). Bacterial morphology of Gram staining was observed using an optical microscope (Nikon Eclipse 50i, Tokyo) and captured using a digital camera (Nikon DS-Fi2, Japan) with the assistance of the ImageView (4.11) software.

To identify strain JHY1, the bacterial strain was first activated by culturing it in LB broth until the optical density (OD_600_) reached 0.6–0.8. At this point, 1 mL of the bacterial suspension was collected and mixed with 567 μL of Tris-EDTA buffer. Subsequently, 15 μL of a 20 mg·mL^−1^ proteinase K solution and 30 μL of 10% SDS solution were added. The mixture was thoroughly vortexed and then incubated in a water bath at 37 °C for 1 h to allow lysis and digestion. After the reaction, 100 μL of 5 M NaCl solution and 80 μL of CTAB/NaCl solution were added, and the mixture was vortexed again. The sample was then incubated at 65 °C for 15 min. Next, an equal volume of chloroform:isoamyl alcohol (24:1) was added, and the mixture was vortexed before being centrifuged to separate the phases. The supernatant was transferred to a new microcentrifuge tube, and 50 μL of isoamyl alcohol was added to aid in phase separation. The sample was centrifuged at 500 rpm for 1 min, and the supernatant was carefully removed. The remaining pellet was washed with 2 mL of 75% ethanol, vortexed, and centrifuged at 500 rpm for 10 min. The supernatant was discarded, and the DNA pellet was resuspended in 20 μL of Tris-EDTA buffer and stored at low temperature for further analysis. The extracted DNA was used for 16S rRNA gene sequencing at GENEWIZ, Inc. (Tianjin, China). The bacterial 16S rDNA fragment was amplified using the primers 27F (5′-AGAGTTTGATCCTGGCTCAG-3′) and 1492R (5′-TACGGCTACCTTGTTACGACTT-3′) [29]. The PCR cycling program consisted of an initial denaturation at 94 °C for 3 min, followed by 30 cycles of denaturation at 94 °C for 45 s, annealing at 55 °C for 30 s, and extension at 72 °C for 90 s. Nucleotide sequencing of the amplified products was performed by GENEWIZ, Inc. (Tianjin, China). After sequencing, the 16S rDNA sequence of strain JHY1 was submitted to GenBank at the National Center for Biotechnology Information (NCBI) and received the accession numbers PV809981-PV809982. The 16S rDNA sequences were compared for homology using the BLAST tool in the NCBI database against the entire GenBank nucleotide database. A phylogenetic tree based on genetic distances was constructed using the neighbor-joining method in MEGA 11 software. The statistical significance of the branching points was assessed using 1000 bootstrap replicates.

### 2.2. Growth Performance and PGP Traits of Strain JHY1

#### 2.2.1. Experimental Design

In this study, dopamine hydrochloride (CAS: 62-31-7) was used as an exogenous DA agent to investigate the effects of different concentrations of NaCl (0, 150, 225, and 300 mM) and DA on the growth of strain JHY1. Referring to previous studies [16,19], a preliminary experiment to promote rice growth was conducted by applying DA concentration gradients of 0, 20, 50, 100, 150, and 200 μM. It was found that the application of 100 μM DA exhibited the most significant growth-promoting effect on rice seedlings. Therefore, 100 μM DA was selected as the optimal concentration for subsequent studies. Additionally, the effect of 100 μM DA on the strain JHY1 was also investigated. Eight treatment groups were established: CK (control), S150, S225, S300, CK + DA, S150 + DA, S225 + DA, and S300 + DA, where “S” represents the experimental groups at the respective concentrations of NaCl (mM) and “DA” represents the addition of exogenous DA at a concentration of 100 μM. Growth curves and PGP traits of strain JHY1 were measured for each treatment group, with all experiments conducted with three biological replicates and three technical replicates.

#### 2.2.2. Growth Performance of Strain JHY1

The strain was inoculated into the LB medium and cultured under the aforementioned eight treatment conditions, with shaking at 180 rpm and incubation at 28 °C. During the first 10 h of cultivation, the optical density of the samples was measured every 2 h. Subsequently, after the initial 10 h period, the measurement frequency was adjusted to every 10 h. The optical density was determined using an ultraviolet spectrophotometer at a wavelength of 600 nm to construct the growth curve.

#### 2.2.3. Determination of PGP Traits of Strain JHY1

When determining the PGP traits of strain JHY1, the strain was first cultured in LB broth at 28 °C and 180 rpm until the OD_600_ reached 0.6–0.8. The bacterial suspension was then used for subsequent experiments.

##### Nitrogen Fixation Capacity

To assess the nitrogen-fixing ability of strain JHY1, the bacterial suspension (2 μL) from the logarithmic growth phase was plated on nitrogen-free solid medium [30]. The formation of transparent zones was observed and recorded after 7 d.

##### IAA Production Capacity

Based on the optimized method of [31], the IAA production capacity of the bacterial strain was measured. The bacterial suspension (100 μL) was inoculated into 10 mL tryptic soy broth (TSB) medium containing 0.5% L-tryptophan and incubated at 28 °C, 180 rpm for 5 d. After incubation, the culture was centrifuged at 9700× *g* for 10 min at 4 °C (high-speed refrigerated centrifuge: Hettich MIKRO 22, Germany), and the supernatant was collected. It should be noted that all centrifugation steps throughout the experiment were carried out using the same model. To each solution of IAA standard and eight treatment groups, 100 μL of 10 mM phosphoric acid and 4 mL of Salkowski reagent were added. After vortexing, the mixtures were incubated in the dark for 30 min, and absorbance was measured at 530 nm. The IAA concentration in the sample was determined using the standard curve method.

##### ACC Deaminase Activity

ACC deaminase activity was measured using the established method [32]. The bacterial suspension (100 μL) was then inoculated into 10 mL of TSB liquid medium and incubated at 28 °C and 160 rpm for 24 h. After incubation, 2 mL of bacterial suspension was centrifuged at 4 °C and 9700× *g* for 10 min, and the cell pellet was washed and resuspended in 2 mL of ADF liquid medium, followed by an incubation at 28 °C and 180 rpm for 4 d. The cells were collected by centrifugation at 4 °C and 9700× *g* for 10 min, washed with pH 7.6 Tris-HCl buffer, vortexed, and resuspended in 600 μL of pH 8.5 Tris-HCl buffer. Subsequently, 30 μL of toluene (analytical grade, 100% concentration) was added, and the mixture was vortexed for 30 s to obtain the cell lysate. For the α-ketobutyric acid standard curve, 0, 50, 100, 150, and 200 μL of the standard solution were pipetted into test tubes and diluted to 200 μL with pH 8.5 Tris-HCl buffer, followed by the addition of 300 μL of 0.2% 2,4-dinitrophenylhydrazine solution. After mixing, the samples were incubated in a 30 °C water bath for 30 min, and the reaction was stopped by adding 2 mL of 2 M NaOH, with color development for 10 min. Absorbance was measured at 540 nm. For the protein standard curve, 0, 40, 80, 120, 160, and 200 μL of bovine serum albumin standard solution were diluted to 200 μL with 0.15 M NaCl solution, and 1 mL of Coomassie Brilliant Blue dye reagent was added. After vortexing and incubating for 5 min, absorbance was measured at 595 nm. In the sample determination, 200 μL of cell lysate was mixed with 20 μL of 0.5 M ACC solution and incubated at 30 °C for 15 min. The reaction was terminated by adding 1 mL of 0.56 M HCl solution, followed by centrifugation at 4 °C and 14,000× *g* for 10 min. The 1 mL supernatant was further mixed with 800 μL of 0.56 M HCl solution, and the absorbance was measured at 540 nm to calculate the α-ketobutyric acid concentration. Additionally, 100 μL of cell lysate was diluted to 200 μL with distilled water, and the absorbance was measured at 595 nm to determine the protein concentration [33]. The specific activity of ACC deaminase was calculated from the standard curve.

##### Siderophore Production Capacity

To assess the siderophore production capacity of strain JHY1, the bacterial suspension (100 μL) was inoculated into 10 mL of modified King’s B (MKB) liquid medium and cultured at 28 °C with shaking at 180 rpm for 48 h. The resulting bacterial suspension was then centrifuged at 14,000× *g* for 10 min at 4 °C. After centrifugation, 1 mL of the supernatant was transferred to a test tube, followed by the sequential addition of 1 mL of Chrome Azurol S colorimetric solution and 20 μL of 5-sulfosalicylic acid solution. The mixture was thoroughly vortexed and allowed to develop color for 5 min. The absorbance of the samples (As) was measured at 700 nm using a spectrophotometer, with distilled water serving as the blank. As a control, the absorbance of the MKB medium (Ar) after the same reaction was also measured. The siderophore unit was calculated by the following formula [(Ar − As)/Ar] × 100% [34,35].

##### Phosphate-Solubilizing Capacity

To determine the phosphate-solubilizing capacity of strain JHY1, the bacterial suspension (100 μL) was inoculated into 10 mL of Pikovskaya liquid medium and cultured at 28 °C and 180 rpm for 5 d. Then, the culture was centrifuged at 14,000× *g* for 10 min at 4 °C, and 500 µL of the supernatant was transferred into a test tube. Subsequently, 500 µL of 10% (*w*/*v*) trichloroacetic acid and 4 mL of a color-developing reagent (prepared by thoroughly mixing 3 M H_2_SO_4_, 2.5% ammonium molybdate solution, 10% ascorbic acid solution, and distilled water in a 1:1:1:2 ratio) were added. After that, the mixture was left to stand at room temperature for 20 min. Finally, the absorbance at 820 nm was measured using a UV spectrophotometer, and the amount of dissolved phosphate was calculated based on a phosphate standard curve prepared with K_2_HPO_4_ [36].

##### Ammonia (NH_3_) Production Capacity

To determine the ammonia production capacity of strain JHY1, the bacterial suspension (100 μL) was inoculated into 10 mL of peptone solution (10 g/L) and then incubated at 28 °C and 160 rpm for 48 h. Afterward, 0.5 mL of the Nessler reagent was added to each test tube, resulting in a color change from brown to yellow, indicating ammonia production. The absorbance of the liquid was measured at a wavelength of 450 nm, and the ammonia-producing capacity of the strain was calculated based on a standard curve prepared with (NH_4_)_2_SO_4_ [37].

##### Biofilm Formation Production Capacity

To determine the biofilm formation production capacity of strain JHY1, the bacterial suspension (100 μL) was inoculated into 10 mL of TSB with 1% (*w*/*v*) glucose and incubated at 28 °C and 160 rpm for 24 h. After incubation, the medium was carefully aspirated, and the test tubes were washed three times with phosphate-buffered saline (PBS, pH 7.3). The samples were then air-dried. Subsequently, 0.1% (*w*/*v*) crystal violet solution was added to each tube for 5 min of staining. Excess dye was removed by aspiration, and the test tubes were rinsed three times with distilled water. The test tubes were dried again, and then 5 mL of 33% (*v*/*v*) glacial acetic acid was added to solubilize the crystal violet. The samples were incubated in a water bath at 37 °C for 30 min to enhance the solubilization process. The absorbance was measured at 570 nm using a spectrophotometer, with distilled water as the reference. Uninoculated TSB medium containing 1% glucose was used as a blank control [38].

##### EPS Production Capacity

To determine the ability of the strain to produce EPSs of strain JHY1, the bacterial suspension (100 μL) was inoculated into 10 mL of an EPS medium and incubated at 28 °C, 180 rpm for 7 d. The bacterial culture was centrifuged at 4 °C and 9700× *g* for 15 min to collect the supernatant, which was then filtered through a 0.45 μm filter membrane for sterilization. A total of 1 mL of the supernatant was placed in the test tube, and the EPSs produced by the bacteria were precipitated to the bottom by slowly adding three times the volume of pre-cooled ethanol under stirring conditions. The mixture was left to stand at 4 °C for 24 h to allow for complete precipitation. Subsequently, the test tubes were placed in a forced-air drying oven at 70 °C and dried for 24 h until the weight remained constant, after which the precipitate was weighed [39].

### 2.3. Pot Experiment

#### 2.3.1. Plant Materials and Treatment Groups

In this study, the rice variety used for the experiments was Jinyou 4011, which was stored in the laboratory. The rice seeds were germinated and cultivated by soaking them in sterile water for 24 h with the water changed every 8 h. Subsequently, the seeds were sterilized with 75% ethanol for 4 min, followed by 5% sodium hypochlorite for 5 min, and then rinsed three times with distilled water. After sterilization, the seeds were placed in sterile petri dishes with sterile water and incubated for 8 d at 28 °C under a 16 h light/8 h dark photoperiod to promote germination. A total of 150 seedlings with uniform growth were selected and transplanted into 15 pots, with 10 seedlings per pot (each pot containing 150 g of soil, which was a 1:1 (*v*/*v*) mixture of nutrient soil and vermiculite). Each treatment contained 3 pots, and the 5 treatments made up a total of 15 pots.

The treatments were as follows: (1) treatment 1 (CK, control), irrigated with 0 mM NaCl; (2) treatment 2 (S, salt stress), irrigated with 225 mM NaCl; (3) treatment 3 (SD, salt stress + DA), irrigated with 225 mM NaCl and 100 μM DA; (4) treatment 4 (SJ, salt stress + strain JHY1), irrigated with 225 mM NaCl and then inoculated with 3 mL of strain JHY1 (OD_600_ = 0.6) the next day; and (5) treatment 5 (SDJ, salt stress + DA + strain JHY1), irrigated with 225 mM NaCl and 100 μM DA and then inoculated with 3 mL of strain JHY1 (OD_600_ = 0.6). All treatment groups were placed in trays and subjected to 30 d of greenhouse cultivation under controlled environmental conditions: 60% relative humidity, 25 °C temperature, a 16 h light/8 h dark photoperiod, and an illumination intensity of 4000 lux. Seedlings were watered twice a week, with 40 mL of solution applied per pot per irrigation. After 30 d of cultivation, the seedlings were harvested. In the pot experiment, the DA treatment group was irrigated with 100 μM DA during each watering event. In contrast, strain JHY1 was applied only once after the transplantation of seedlings and was not re-inoculated.

#### 2.3.2. Measurement of Rice Growth Parameters

Photographs were taken to document the growth status of rice seedlings 30 d after transplantation. The plants were then carefully uprooted to measure the root length, fresh weight (FW), dry weight (DW), and turgid weight (TW). Using these measurements, the RWC was calculated with the following formula: RWC = [(FW − DW)/(TW − DW)] × 100% [40].

#### 2.3.3. Measurement of Chlorophyll Content and Photosynthetic Rate

Chlorophyll was extracted from 0.1 g of rice leaves under dark conditions using 10 mL of 95% ethanol for 48 h, following established methods [41]. The optical densities at 649 nm (OD_649_) and 665 nm (OD_665_) were measured with a UV spectrophotometer to calculate chlorophyll a and b concentration content. The net photosynthetic rate (Pn) was measured at 11:00 AM on a sunny day using a portable photosynthesis system (LI-6400XT, Lincoln, NE, USA) [42].

#### 2.3.4. Measurement of Antioxidase and Osmotic Substances

The enzyme solution for antioxidant enzyme activity determination was prepared by grinding 0.2 g of the rice leaves thoroughly in 3 mL of pre-cooled PBS (pH 7.8), followed by centrifugation at 4 °C and 9700× *g* for 10 min. The supernatant was then stored at 4 °C pending measurement and used sequentially for the determination of four antioxidant enzyme activities: SOD, peroxidase (POD), ascorbate peroxidase (APX), and catalase (CAT), using established methods from previous studies [43]. The storage period did not exceed 6 h to maintain enzyme stability.

The malondialdehyde (MDA) level in rice, an indicator of lipid peroxidation, was determined using the thiobarbituric acid (TBA) method [44]. To determine the MDA content, 0.2 g of fresh rice leaves was weighed and ground thoroughly in a pre-cooled mortar with 3 mL of PBS (pH 7.8) on ice. The mixture was then centrifuged at 4 °C and 9700× *g* for 10 min. In total, 1 mL of the supernatant was mixed thoroughly with 2 mL of 0.6% TBA solution (prepared in 10% trichloroacetic acid) and heated at 95 °C for 15 min to complete the color reaction. The mixture was rapidly cooled to room temperature. The absorbance of the sample was measured at 450 nm, 600 nm, and 532 nm using a spectrophotometer. The concentration of MDA was calculated using the following formula: C = 6.4 × (OD_532_ − OD_600_) − 0.56 × OD_450_.

To measure the proline content in rice, the ninhydrin method [45] was adopted. The fresh rice leaves (0.2 g) were ground in 2 mL of 3% sulfosalicylic acid solution and immediately centrifuged at 4 °C for 10 min at 10,000× *g*. The supernatant (1 mL) was mixed with 1 mL of glacial acetic acid and 1 mL of acidic ninhydrin solution in a test tube and heated in a boiling water bath for 10 min. The mixture was then cooled in an ice bath. Subsequently, 2 mL of toluene was added to the test tube. After standing for 5 min, the upper toluene layer was taken to measure its absorbance at 520 nm using a spectrophotometer. The proline content was determined using an L-proline standard curve.

To determine the soluble protein content in rice, the Coomassie blue staining method [33] was used. The fresh leaves (0.2 g) were weighed and ground in a pre-cooled mortar with 3 mL of PBS (pH 7.8) on ice. The ground mixture was centrifuged at 4 °C and 14,000× *g* for 15 min to obtain the supernatant. The supernatant (0.2 mL) was mixed with 1 mL of Coomassie Brilliant Blue G-250 reagent, and the mixture was allowed to react for 3 min. The absorbance was measured at 595 nm using a spectrophotometer. A standard curve was prepared using bovine serum albumin to calculate the total soluble protein content in the sample.

To assess the soluble sugar content in rice, the anthrone method [46] was employed. The supernatant preparation process was the same procedure as in the soluble protein determination. The supernatant (0.5 mL) was mixed with 2.5 mL of anthrone reagent and heated in a water bath at 100 °C for 5 min. After rapid cooling, the absorbance was recorded at 620 nm using a spectrophotometer. The concentration of soluble sugar in the sample was determined based on the glucose standard curve.

#### 2.3.5. Assessment of Soil Fertility Levels

To assess soil fertility, the levels of nitrogen (ammonium and nitrate nitrogen) and available phosphorus [47], as well as soil enzyme activity, were measured based on methods established in previous studies [30,48].

To determine soil urease activity, 2 g of soil was weighed into a conical flask. Subsequently, 1 mL of toluene was added, and the mixture was vortexed for 15 min. Then, 10 mL of urea solution (10%, *w*/*v*) and 20 mL of citrate buffer (pH 6.7) solution were added. The flask was incubated in a constant-temperature shaker at 37 °C and 150 rpm for 24 h. After incubation, the mixture was filtered using qualitative filter paper, and 1 mL of the filtrate was transferred to a test tube. To this, 4 mL of phenol sodium and 3 mL of 0.9% sodium hypochlorite solution were added. The solution was left to stand for 20 min to develop color, and the absorbance of the colored solution was immediately measured at 578 nm using a spectrophotometer. To determine soil sucrase activity, 2 g of soil was weighed into a conical flask. To this, 15 mL of sucrose solution (8%, *w*/*v*), 5 mL of PBS (pH 5.5), and 5 mL of toluene were added. The flask was incubated in a constant-temperature shaker at 37 °C and 150 rpm for 24 h. After incubation, the mixture was filtered using qualitative filter paper, and 1 mL of the filtrate was transferred to a test tube. Then, 3 mL of DNS reagent was added, and the mixture was vortexed. The solution was then subjected to a boiling water bath for 5 min to develop color. After cooling to room temperature, the absorbance of the colored solution was measured at 508 nm using a spectrophotometer. To determine soil alkaline phosphatase (ALP) activity, 2 g of soil was weighed into a conical flask and mixed with 0.5 mL of toluene solution. After thorough mixing for 15 min, 10 mL of phenyl phosphate sodium solution was added. The flask was incubated in a constant-temperature shaker at 37 °C and 150 rpm for 24 h. After incubation, the mixture was filtered using qualitative filter paper, and 2 mL of the filtrate was transferred to a test tube. Next, 0.5 mL of borate buffer solution (pH 9.0), 0.3 mL of 2.5% potassium ferricyanide solution, and 0.3 mL of 0.5% 4-aminoantipyrine solution were added sequentially. The solution was diluted to 10 mL with distilled water, thoroughly mixed, and left to stand for 20 min until the color stabilized. The absorbance of the colored solution was then measured at 570 nm using a spectrophotometer. To determine soil protease activity, 2 g of soil was weighed into a conical flask and mixed with 10 mL of 1% (*w*/*v*) casein solution and 0.5 mL of toluene solution. The mixture was incubated in a constant-temperature shaker at 30 °C and 150 rpm for 24 h. After incubation, the mixture was filtered using qualitative filter paper. Then, 1 mL of the filtrate, 0.5 mL of sulfuric acid solution (0.1 M), and 0.5 mL of sodium sulfate solution (20%, *w*/*v*) were added, and the mixture was left to stand for 10 min. The solution was centrifuged at 25 °C and 3500× *g* for 15 min, and the supernatant was collected. All of the supernatant was collected, and 1 mL of ninhydrin reagent was added, followed by heating in a boiling water bath for 10 min. After dilution and cooling, the absorbance of the diluted colored solution was measured at 560 nm using a spectrophotometer. Enzyme activities were calculated from standard curves.

#### 2.3.6. Determining Na^+^ and K^+^ Concentrations in Plants and Soil

Rice rhizosphere soil was air-dried, sieved, and two ions (Na^+^ and K^+^) were extracted using 0.005 M diethylenetriaminepentaacetic acid (DTPA). The soil (5 g) was mixed with 25 mL of DTPA solution, shaken at 28 °C and 180 rpm for 2 h, then filtered using qualitative filter paper. The filtrate was adjusted to 50 mL and stored at 4 °C. For rice samples, the concentrations of the two ions were measured separately in the shoots, roots, and whole plants. Specifically, dried material (0.1 g) was digested with 5 mL of a nitric acid–perchloric acid mixture (3: 1 *v*/*v*) after overnight soaking, followed by heating at 200 °C for 3 h. The cooled digest was filtered using qualitative filter paper, adjusted to 10 mL, and stored at 4 °C. Na^+^ and K^+^ concentrations were determined via ICP-OES at 589.5 nm and 766.4 nm, respectively [49].

#### 2.3.7. Analysis of Rhizosphere Microbial Community

When uprooting the rice plants, the rhizosphere soil was gently shaken off and collected for the analysis of rhizosphere microbial communities. First, DNA was extracted from the rhizosphere soil samples stored at −20 °C. The extracted DNA was then used for PCR amplification of 16S rDNA, followed by sequencing using the Illumina HiSeq 2500 system. The results of principal coordinates analysis (PCoA) were performed using R packages to compare the degree of similarity among treatments in terms of bacterial species diversity. Multisample rarefaction curves and multisample Shannon curves were obtained from the BMKcloud platform. Species distribution charts that represent the phylum and genus levels illustrated the relative abundance of rhizosphere soil microbial communities across the different treatments. Heatmaps were generated using the heatmap package in RStudio. The linear discriminant analysis effect size (LEfSe) method (linear discriminant analysis, LDA score > 3.5) was employed to identify key bacterial biomarkers that significantly differed in the rhizosphere microbial community between different groups. A redundancy analysis (RDA) explored the relationships between rhizosphere microbial abundance and environmental factors [50]. A Spearman correlation analysis was used to evaluate the relationships among the top 50 bacterial genera in relative abundance in the rhizosphere soil of rice (*p* < 0.05), and the results are presented as a correlation network diagram.

### 2.4. Data Analysis

The experimental data are presented as mean ± standard deviation of three independent replicates. A statistical analysis of the means was performed using SPSS 26. Differences between groups were assessed by one-way or two-way analysis of variance (ANOVA). Duncan’s multiple range test was applied to indicate the significance of group differences, with different lowercase letters (a, b, c, d, e) denoting statistically significant differences at the *p* < 0.05 level. Data analysis and histogram generation were performed using R 4.3.3. Partial least squares path modeling (PLS-PM) was used to assess the combined effects of plant growth, soil nutrients, Na^+^-K^+^ balance in plant or soil, and stress response indicators on plant performance under different treatments.

## 3. Results

### 3.1. Characterization of PGPR Under Salt Stress

**Figure 1:**
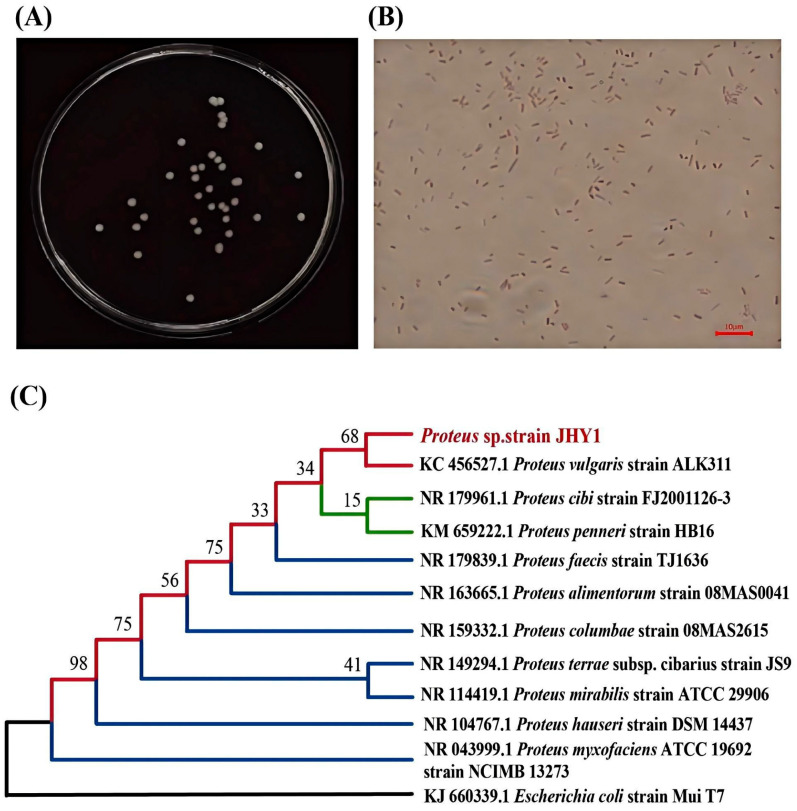
Salt-tolerant, plant-growth-promoting strain JHY1. (**A**) Colony morphology. (**B**) Gram staining observed under a microscope. (**C**) Phylogenetic tree showing the relationship of strain JHY1 with closely related species.

On the agar plates spread with the diluted strain JHY1 bacterial culture, the colonies appeared smooth, circular, and cream-colored (Figure 1A). A microscopic observation of strain JHY1 after Gram staining revealed that the bacteria were short, rod-shaped, and stained red, confirming the strain as Gram-negative (Figure 1B). A phylogenetic analysis based on 16S rDNA sequencing showed that strain JHY1 shared 99% sequence similarity with *Proteus vulgaris* strain ALK311 (KC 456527.1). A phylogenetic tree illustrating the phylogenetic relationships of strain JHY1 was presented in Figure 1C. This suggested that strain JHY1 was closely related to *Proteus vulgaris* strain ALK311 (KC 456527.1) and belonged to the genus *Proteus* within the phylum Proteobacteria.

### 3.2. Determination of Growth Curve and Growth-Promoting Indicators of Strain JHY1

**Figure 2:**
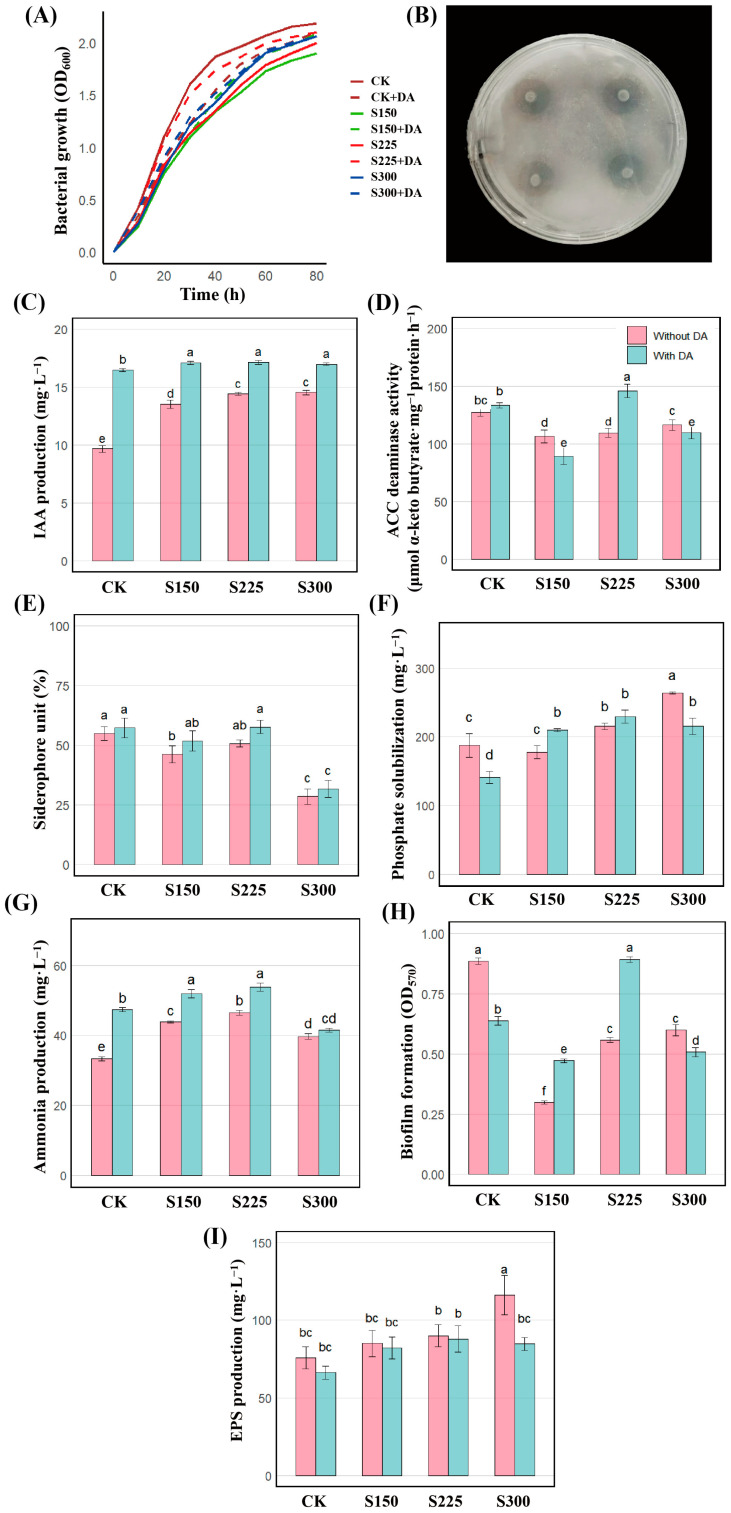
Growth performance and PGP traits of strain JHY1 under different NaCl concentrations (NaCl, CK: 0 mM; S150: 150 mM; S225: 225 mM; S300: 300 mM) with or without DA (DA, 100 μM). In the bar charts of (**C**–**I**), pink bars represent the groups without DA treatment at the corresponding salt concentrations, while blue bars represent the groups with DA treatment added at the corresponding salt concentrations. (**A**) Growth curves. (**B**) Clear halos formed on nitrogen-free medium, indicating nitrogen fixation. (**C**) IAA production. (**D**) ACC deaminase activity. (**E**) Siderophore production. (**F**) Phosphate solubilization. (**G**) Ammonia production. (**H**) Biofilm formation. (**I**) EPS production. Values are the means ± standard deviations of three replicates. Different letters indicate significant differences between groups according to ANOVA and Duncan’s test (*p* < 0.05).

The nitrogen-fixing ability of strain JHY1 was evaluated using the transparent zone method. As shown in Figure 2B, a distinct transparent zone was observed on the plates inoculated with strain JHY1 after 7 d, indicating that strain JHY1 possessed nitrogen-fixing activity.

Under normal conditions (0 mM NaCl), strain JHY1 exhibited a typical growth curve (Figure 2A). The strain reached the exponential phase with an OD_600_ value of 0.432 at 6 h and entered the stationary phase with an OD_600_ value of 2.069 by 60 h. However, compared to the control, the growth of strain JHY1 was inhibited under 150, 225, and 300 mM NaCl, with the exponential phase OD_600_ decreasing to 0.247, 0.298, and 0.278 at 6 h (*p* < 0.05), respectively, and the stationary phase OD_600_ declining to 1.729, 1.790, and 1.907 (*p* < 0.05), respectively. At 150, 225, and 300 mM NaCl, the addition of DA alleviated the inhibitory effects of salt, resulting in an increase in OD values. The strain JHY1 in the group with DA grew better than that in the group without DA at each salt concentration. From 10 h to 80 h, the growth differences between the groups with and without DA under 0 (CK) and 225 mM salt stress were significant. These results suggested that DA might enhance the salt tolerance of strain JHY1 by improving its metabolic adaptation to high salinity.

Figure 2C shows that strain JHY1 exhibited high IAA production under different NaCl concentrations (0, 150, 225, and 300 mM), with the lowest production observed at 0 mM and a gradual increase at 150 and 225 mM. The IAA production of strain JHY1 at 300 mM NaCl was comparable to that at 225 mM. Importantly, the IAA production of strain JHY1 by the strain did not decrease under salt stress. Under various salt concentrations (0, 150, 225, and 300 mM) with DA, the production of IAA was further enhanced compared to the groups without DA, with increases of 73.19%, 24.78%, 20.75%, and 16.16%, respectively. Figure 2D showed that under 150 mM NaCl concentrations, ACC deaminase activity was inhibited compared to the control group, with reductions of 14.73%. Under salt concentrations of 150 and 300 mM with DA, the ACC deaminase activity showed a decreasing trend compared with the group without DA. However, at the salt concentration of 225 mM, which was the actual treatment level for rice, the ACC deaminase activity of the strain with DA showed an upward trend compared to the group without DA.

As shown in Figure 2E, under salt stress at concentrations of 150 and 300 mM NaCl, the production of siderophores by the bacteria decreased by 15.82% and 48.10% compared to the control group, respectively. Under different salt concentrations (0, 150, 225, and 300 mM) in the presence of DA, siderophore production showed no significant differences compared to the groups without DA. Figure 2F showed that the strain’s phosphate-solubilizing ability showed no significant change under 150 mM NaCl compared to the control. However, under 300 mM NaCl, it significantly increased by 40.88%, with dissolved phosphate reaching 264 mg/L, highlighting its improved performance under high salinity. As shown in Figure 2G, under salt concentrations of 150, 225, and 300 mM NaCl, ammonia production increased by 31.62%, 39.25%, and 18.90%, respectively, compared to the control group, reaching its highest level (46.48 mg/L) at 225 mM NaCl. The addition of DA further enhanced ammonia production by 41.86%, 17.30%, and 15.82% at 0, 150, and 225 mM NaCl, respectively, compared to the corresponding groups without DA, while at 300 mM NaCl, no notable difference was observed.

As shown in Figure 2H, at salt concentrations of 150, 225, and 300 mM, the biofilm content of the strain decreased significantly by 66.25%, 37.02%, and 32.27%, respectively, compared to the control group. At salt concentrations of 0 and 300 mM with DA, the biofilm content was reduced by 27.99% and 15.33% compared to the corresponding groups without DA. However, at salt concentrations of 150 and 225 mM with DA, the biofilm content increased by 57.86% and 60.04%, respectively, compared to the groups without DA. Figure 2I demonstrated that as the salt stress concentration increased, the production of EPSs by the bacteria showed an upward trend. Specifically, there was no significant difference compared to the control group at salt concentrations of 150 and 225 mM. However, at a salt concentration of 300 mM, the production of EPSs by the bacteria increased significantly by 53.67%. In the presence of DA at different salt concentrations, there was no significant difference in EPS production among the groups when compared to the control group.

The above results show that under salt stress, strain JHY1 had PGP traits such as IAA production, ACC deaminase activity, phosphate solubilization, siderophore production, ammonia production, biofilm formation, and EPS production. After DA treatment at different salt concentrations, the corresponding PGP indicators will increase or decrease. It is worth noting that below 300 mM salt concentration, the IAA production ability of the strain was significantly enhanced.

### 3.3. Effects of Individual and Co-Application of DA and Strain JHY1 on Growth Parameters of Rice Under Salt Stress

**Figure 3:**
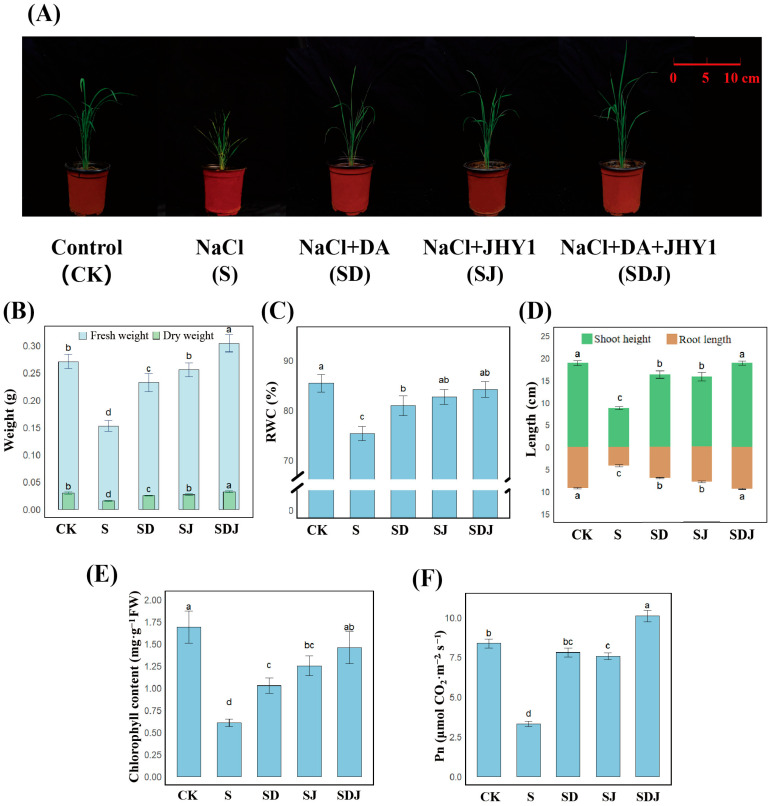
Growth parameters of rice across treatment groups. (**A**) Morphological appearance of rice plants 30 d after transplantation. (**B**) Fresh weight and dry weight. Light blue bars represent the measured fresh weight of rice, and light green bars represent the measured dry weight. (**C**) RWC. (**D**) Shoot height and root length. Green bars represent the height of the above-ground part (shoot), and brown bars represent the length of the below-ground part (root length). (**E**) Chlorophyll content. (**F**) Pn. RWC: relative water content. Pn: net photosynthetic rate. CK: 0 mM NaCl. S: 225 mM NaCl. SD: 225 mM NaCl + 100 μM DA. SJ: 225 mM NaCl + strain JHY1. SDJ: 225 mM NaCl + 100 μM DA + strain JHY1. Values are the means ± standard deviations of three replicates. Different letters indicate significant differences between groups according to ANOVA and Duncan’s test (*p* < 0.05).

The morphological characteristics of rice in different treatment groups after being cultured for 30 d are shown in Figure 3A. As shown in Figure 3B–F, irrigation with 225 mM saline water significantly inhibited the fresh weight (by 43.66%), dry weight (by 48.81%), RWC (by 11.75%), shoot height (by 48.47%), root length (by 54.41%), chlorophyll content (by 64.02%), and Pn (by 60.53%) of rice plants. Compared with the salt-treated group, rice inoculated with strain JHY1 exhibited increased fresh weight (by 67.61%), dry weight (by 76.67%), RWC (by 9.77%), shoot height (by 61.71%), root length (by 86.03%), chlorophyll content (by 106.10%), and Pn (by 129.09%). When compared to the salt-treated group, the combined application of DA and strain JHY1 resulted in a more substantial increase in fresh weight (by 99.38%), dry weight (by 113.33%), RWC (by 11.7%), shoot height (by 93.44%), and root length (by 123.08%). Notably, this combination significantly enhanced chlorophyll content (by 139.84%) and Pn (by 206.06%). Compared with the control group, the combined application of DA and strain JHY1 resulted in a significant increase in Pn (by 20.81%), while no significant differences were observed in other growth parameters. These results demonstrated that the combined application of DA and strain JHY1 was more effective in promoting rice growth under salt stress.

### 3.4. Effects of Individual and Co-Application of DA and Strain JHY1 on Antioxidant Enzymes and Osmotic Substances of Rice Under Salt Stress

**Figure 4:**
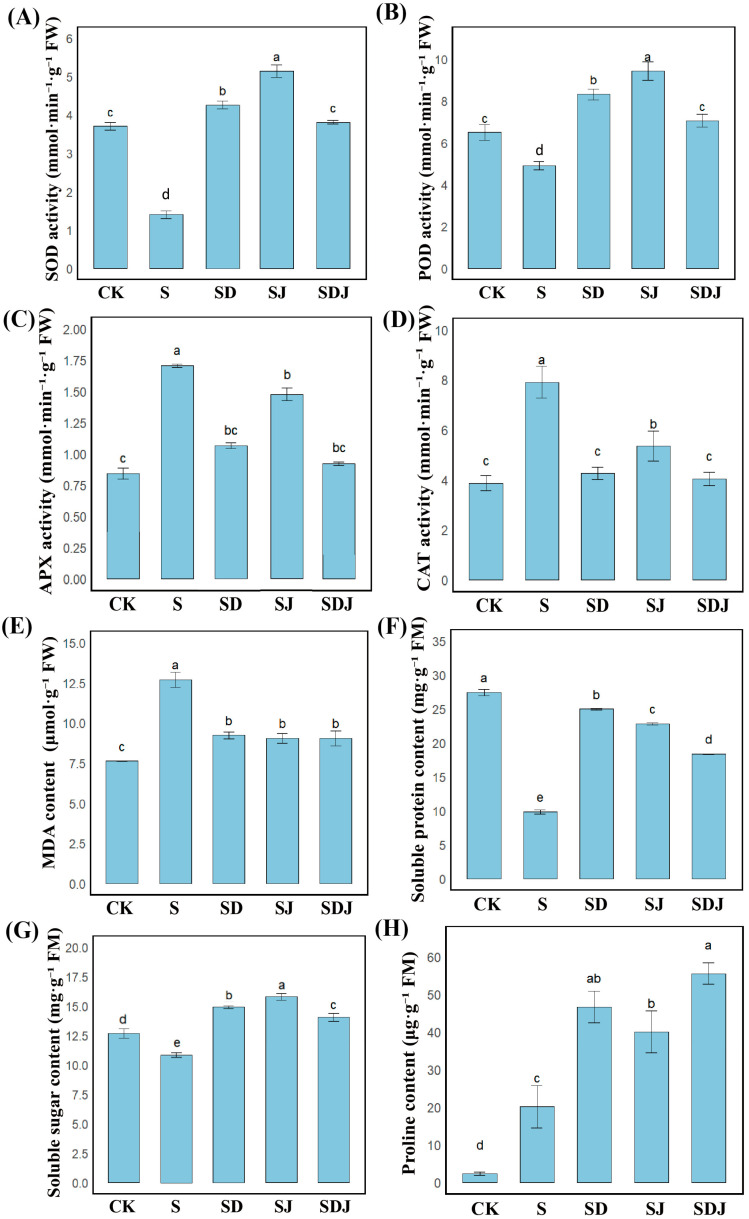
Antioxidant enzyme activities, lipid peroxidation, and osmotic balance indicators in rice. (**A**–**D**) Activities of antioxidant enzymes: (**A**) SOD activity, (**B**) POD activity, (**C**) APX activity, and (**D**) CAT activity in rice leaves. (**E**) MDA content. (**F**,**G**) Osmotic balance. (**F**) Soluble protein content. (**G**) Soluble sugar content. (**H**) Proline content. CK: 0 mM NaCl. S: 225 mM NaCl. SD: 225 mM NaCl + 100 μM DA. SJ: 225 mM NaCl + strain JHY1. SDJ: 225 mM NaCl + 100 μM DA + strain JHY1. Values are the means ± standard deviations of three replicates. Different letters indicate significant differences between groups according to ANOVA and Duncan’s test (*p* < 0.05).

Figure 4A–D showed that salt stress decreased SOD and POD levels by 62.20% and 24.57%, respectively, while APX and CAT increased by 55.20% and 46.25%, respectively. Notably, in the group inoculated with strain JHY1, the activities of SOD and POD significantly increased by 267.41% and 92.44%, respectively, while the activities of APX and CAT were decreased by 21.40% and 32.32%, respectively. In the group treated with the combined application of DA and strain JHY1, the activities of SOD and POD significantly increased by 204.37% and 69.69%, respectively. The decreases in APX and CAT activities were more pronounced, with reductions of 52.78% and 51.07%, respectively, approaching the levels of the control group. To assess lipid peroxidation in rice, the content of MDA was measured. As shown in Figure 4E, the MDA level in the salt stress group increased by 66.47% compared to the control group. The application of DA, strain JHY1, and their combination all reduced MDA levels compared to the salt stress group by 27.24%, 28.65% and 28.69%, respectively. These results indicated that the trend of antioxidant enzyme levels in the rice treated with the combination of DA and strain JHY1 was closer to that of the control group, and the level of MDA could be significantly reduced.

As shown in Figure 4H, salt stress significantly increased proline content by 766.38% compared with the control group, indicating that proline played an indispensable role in the response to salt stress. The application of strain JHY1 alone increased proline content by 99.50% compared with the salt-stressed group, while the combined application of DA and strain JHY1 further enhanced proline content by 176.67%. Furthermore, Figure 4F showed that salt stress decreased soluble protein content by 64.12% compared with the control group. However, the application of strain JHY1 and the combined application of DA and strain JHY1 effectively alleviated this decline, increasing soluble protein content by 131.77% and 86.36%, respectively, compared with the salt-stressed group. As indicated in Figure 4G, salt stress reduced soluble sugar content by 14.42% compared with the control group. The application of strain JHY1 and the combined application of DA and strain JHY1 both showed an increasing trend in soluble sugar content compared with the salt-stressed group. The above results showed that the combined application of DA and strain JHY1 could significantly increase the content of osmotic substances in rice plants, especially achieving the best intergroup effect on the increase in proline.

### 3.5. Effects of Individual and Co-Application of DA and Strain JHY1 on Soil Nutrient Dynamics and Enzymatic Activity in Rice Under Salt Stress

**Figure 5:**
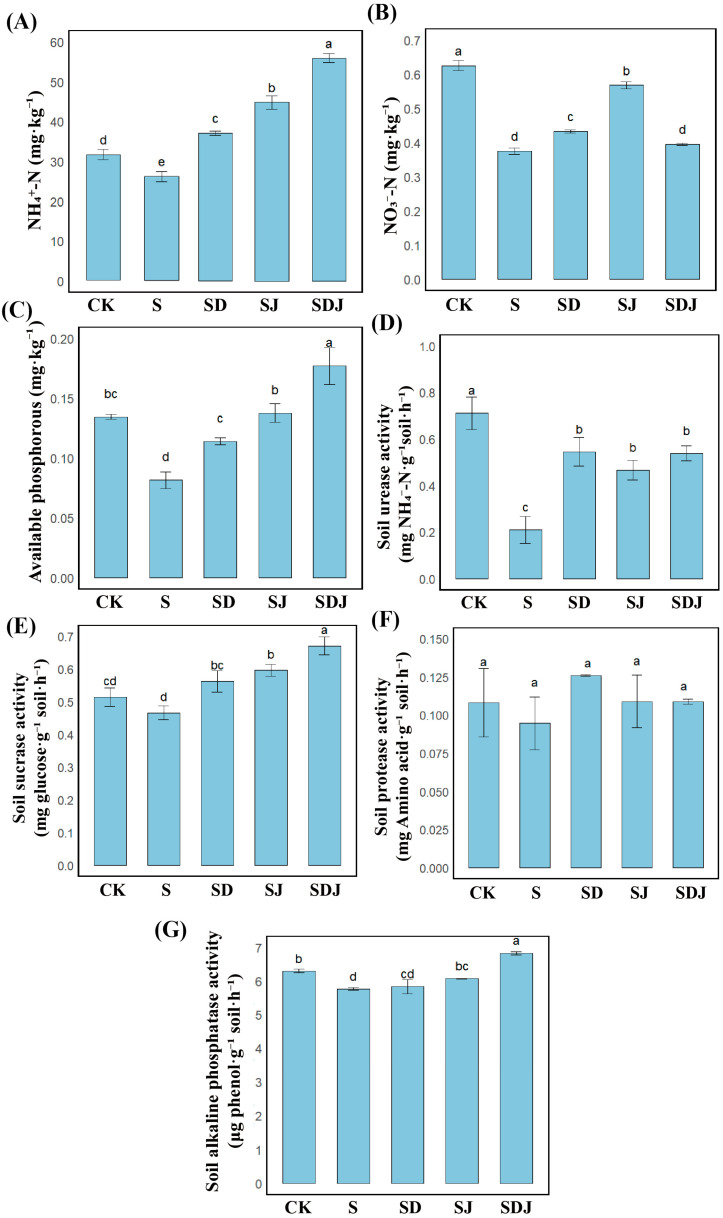
Soil fertility parameters under different treatments. (**A**) NH_4_^+^-N. (**B**) NO_3_^−^-N. (**C**) Available phosphorus. (**D**) Soil urease activity. (**E**) Soil sucrase activity. (**F**) Soil protease activity. (**G**) Soil alkaline phosphatase activity. CK: 0 mM NaCl. S: 225 mM NaCl. SD: 225 mM NaCl + 100 μM DA. SJ: 225 mM NaCl + strain JHY1. SDJ: 225 mM NaCl + 100 μM DA + strain JHY1. Values are the means ± standard deviations of three replicates. Different letters indicate significant differences between groups according to ANOVA and Duncan’s test (*p* < 0.05).

As shown in Figure 5A–C, compared to the control group, the levels of NH_4_^+^-N and NO_3_^−^-N in the salt-treated soil were significantly reduced by 40.13% and 17.14%, respectively, resulting in a significant decrease in available phosphorus by 39.37%. As shown in Figure 5D–G, in the salt-treated group, ALP activity was 8.43% lower and soil urease activity was 70.51% lower than in the control group. In contrast, protease and sucrase activities showed no significant differences. Combined DA and strain JHY1 treatment increased the levels of both nitrogen forms and available phosphorus, as well as ALP and soil urease activities. When DA and strain JHY1 were applied together, NH_4_^+^-N levels increased by 51.69%, soluble phosphorus by 217.04%, sucrase activity by 44.00%, and ALP activity by 18.45%, all exceeding the levels of the control group. The combined treatment also showed significantly greater improvements than either treatment alone. These results indicated that the combined treatment was more effective than the individual strategy in restoring soil nitrogen and phosphorus levels and increasing soil enzyme activities, thereby improving soil fertility.

### 3.6. Na^+^ and K^+^ Ionic Regulation Under Salt Stress in Rice and Soil

**Figure 6:**
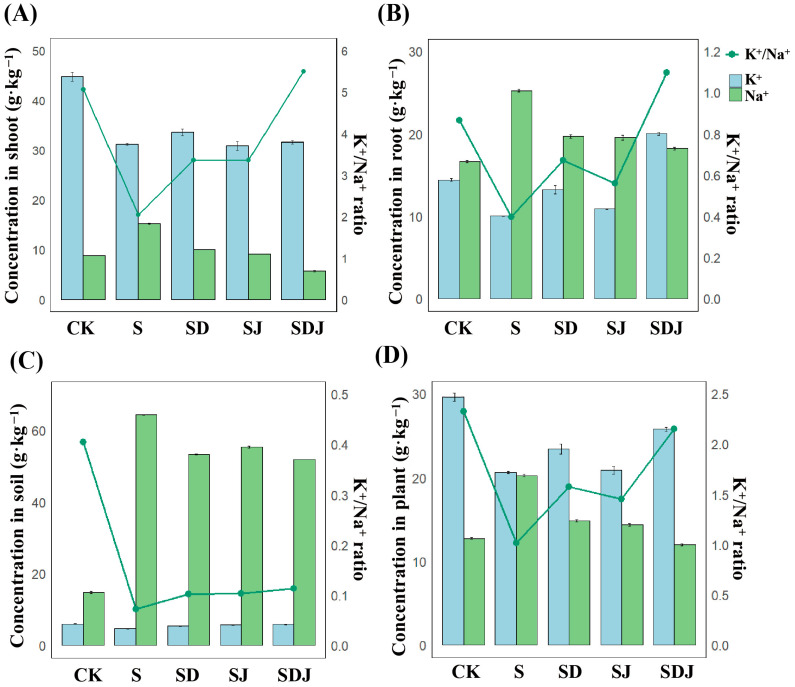
Na^+^ and K^+^ concentration and K^+^/Na^+^ ratio. (**A**) In shoot of rice. (**B**) In root of rice. (**C**) In rice rhizosphere soil. (**D**) In whole plant. Blue bars represent the K^+^ concentration in each component, and green bars represent the Na^+^ concentration. The concentrations of both ions should be read according to the left y-axis values and units. The green line represents the K^+^/Na^+^ ratio, which should be read according to the right y-axis. CK: 0 mM NaCl. S: 225 mM NaCl. SD: 225 mM NaCl + 100 μM DA. SJ: 225 mM NaCl + strain JHY1. SDJ: 225 mM NaCl + 100 μM DA + strain JHY1. Values are the means ± standard deviations of three replicates.

The results (Figure 6A–D) showed that in all plant parts, the Na^+^ concentration was significantly higher in the salt-treated group. The K^+^ content in the shoot aligned with the overall and root K^+^ content trends, where the CK group exhibited higher K^+^ levels than the salt-stressed treatments. Soil bioavailable Na^+^ levels increased by 333.67% due to exogenous NaCl application, while Na^+^ levels in rice increased by 59.02%, with roots and shoots increasing by 52.71% and 72.79%, respectively. The K^+^/Na^+^ ratio was lower in the salt-treated group compared with other treatment groups, suggesting that Na^+^ may have caused some degree of damage to the rice. The strain JHY1 inoculation and DA application treatments reduced Na^+^ accumulation in rice by 29.17% and 26.80%, respectively, while the combined treatment showed the best effect, reducing Na^+^ uptake by 40.88% (Figure 6C). Additionally, DA application increased K^+^ concentration in plants, with the K^+^/Na^+^ ratio improving by 13.50% in the DA treatment group and 25.03% in the combined treatment group. The results showed that the combined treatment of DA and strain JHY1 increased the K^+^/Na^+^ ratio in plants and soil under salt stress, which was more effective than a single treatment.

### 3.7. Rhizosphere Microbial Community in the Soil of the Strain JHY1-Treated Group and the DA-JHY1 Co-Treated Group

**Figure 7:**
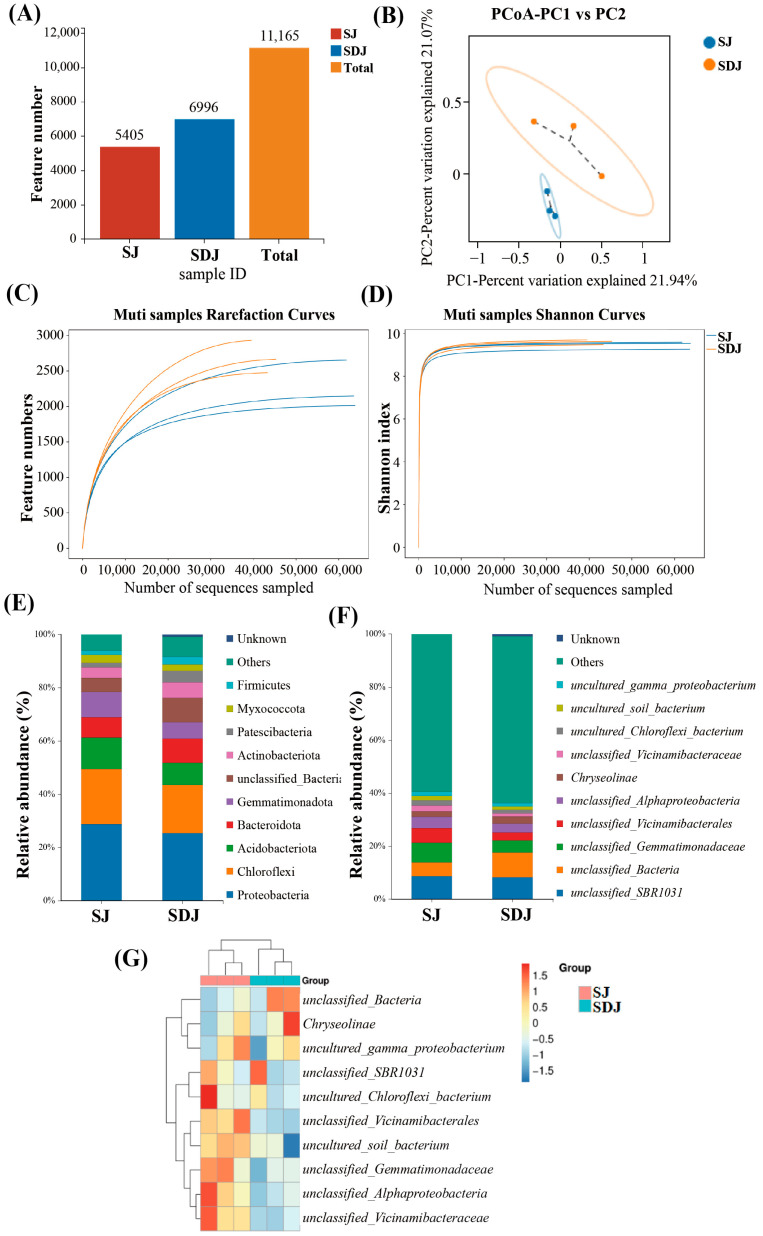
Diversity and structure of rhizosphere microbial communities. (**A**) Feature number. (**B**) PCoA analysis. (**C**) Rarefaction curves. (**D**) Shannon curves. (**E**) Relative abundance (phylum). (**F**) Relative abundance (genus). (**G**) Heatmap clustering. SJ: 225 mM NaCl + strain JHY1. SDJ: 225 mM NaCl + 100 μM DA + strain JHY1.

To investigate the effects of DA application on the rhizosphere microbial community of rice inoculated with the growth-promoting strain JHY1, the soil microbial communities from treatments with strain JHY1 alone and the combined application of DA and strain JHY1 were analyzed. The feature counts for the microbial communities in the SJ and SDJ groups were 5405 and 6996, respectively, with a total feature count of 11,165 (Figure 7A). Based on the Shannon index, the group SDJ showed higher microbial abundance and diversity. The rarefaction curves and Shannon index stabilized with sequencing depth, indicating that the measured data adequately reflected the true community abundance (Figure 7C,D). At the phylum level (Figure 7E), the relative abundance of Firmicutes, Actinobacteria, and Bacteroidetes increased by 79.49%, 41.12%, and 20.67%, respectively, in the DA-JHY1 co-treated group, whereas Proteobacteria and Acidobacteria decreased by 11.57% and 30.21%, respectively. Similarly, at the genus level (Figure 7F), the relative abundance of *Chryseolinea* and *unclassified_Bacteria* increased by 27.21% and 80.54%, respectively, in the DA-JHY1 co-treated group. A heatmap analysis (Figure 7G) and PCoA (Figure 7B) revealed a clear distinction in the microbial communities between the JHY1-treated group and the DA-JHY1 co-treated group, indicating that DA application significantly altered the rhizosphere microbial community of rice.

**Figure 8:**
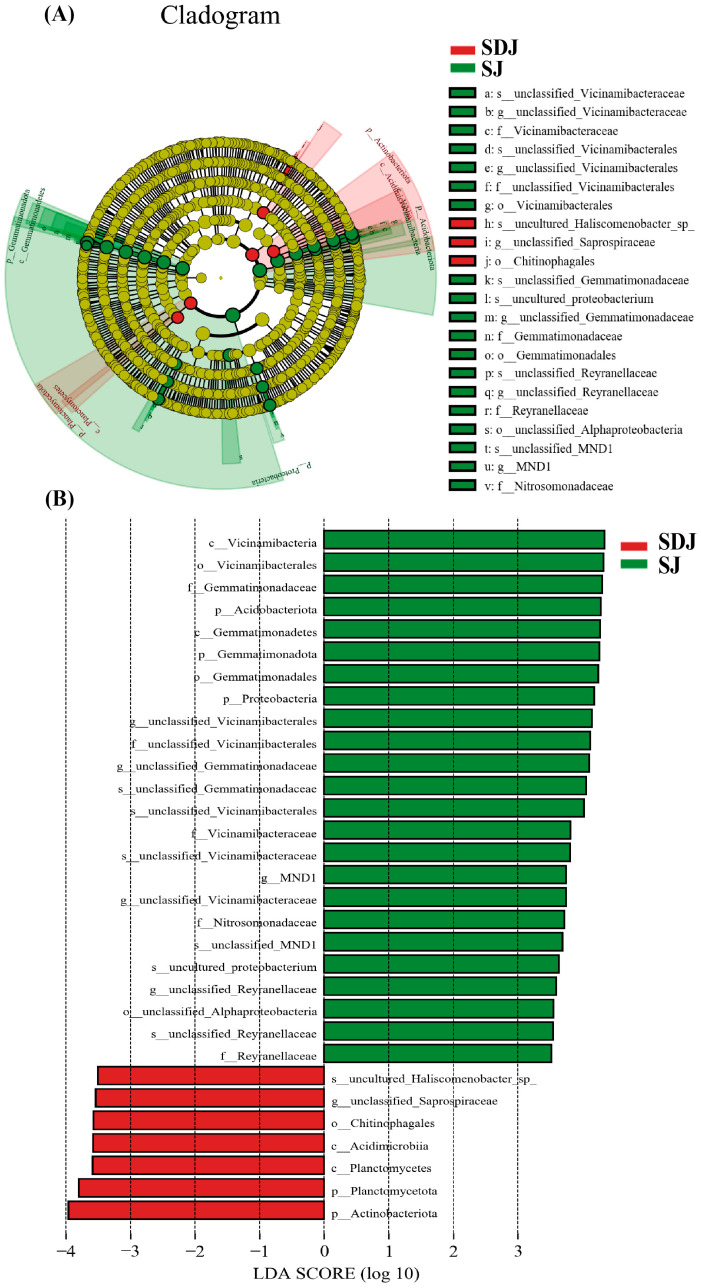
LEfSe analysis of rhizospheric microorganism. (**A**) The taxonomic diversity and evolutionary relationships of the soil microbial community from the phylum to species level. (**B**) LDA score bar chart. SJ: 225 mM NaCl + strain JHY1. SDJ: 225 mM NaCl + 100 μM DA + strain JHY1.

**Figure 9:**
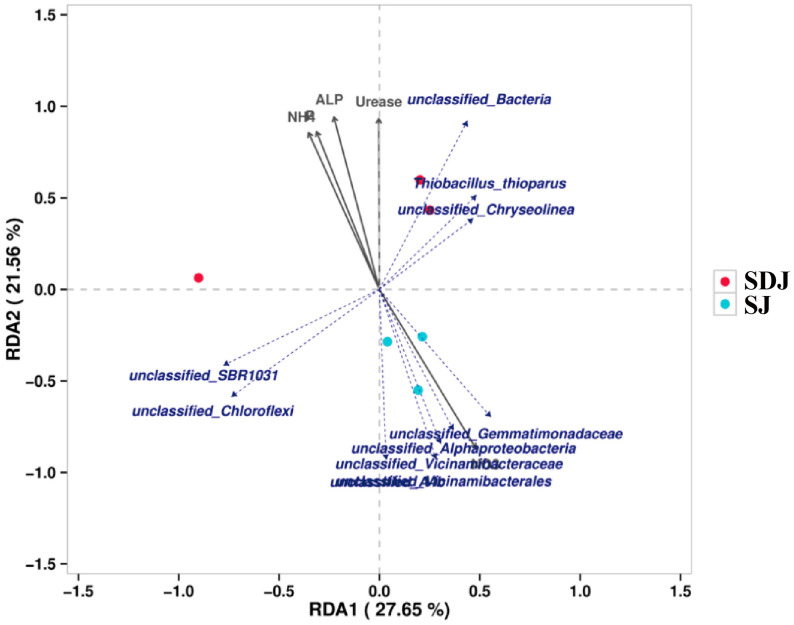
RDA analysis of correlations between environmental factors (NH4: ammonium nitrogen content; NO3: nitrate nitrogen content; P: available phosphorus content; ALP: alkaline phosphatase activity; Urease: urease activity) and the rhizosphere microbial abundance. SJ: 225 mM NaCl + strain JHY1. SDJ: 225 mM NaCl + 100 μM DA + strain JHY1.

**Figure 10:**
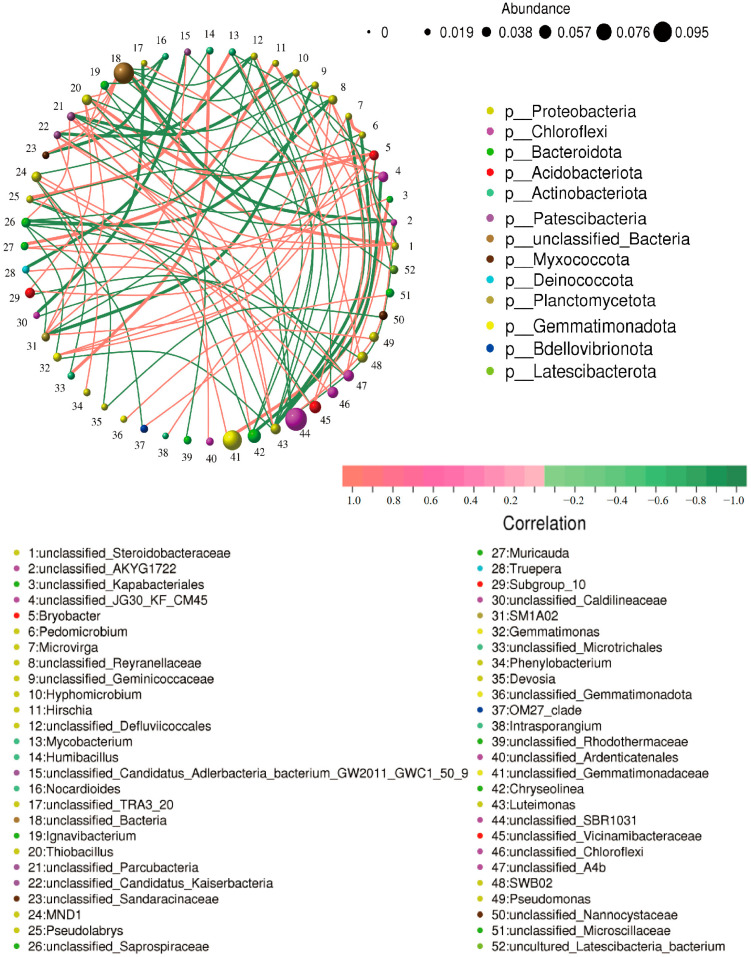
Correlation network of rice rhizosphere bacteria. Each node represents a bacterial genus. The size of the node reflects the abundance of the genus, and the color indicates the phylum to which the genus belongs. Lines between nodes represent the correlation relationships between bacterial genera. The thickness and darkness of the lines indicate the strength of the correlation: thicker and darker lines represent stronger correlations, while thinner and lighter lines represent weaker correlations. Red lines indicate positive correlations, and green lines indicate negative correlations.

The cladogram (Figure 8A) illustrated the taxonomic diversity and evolutionary relationships of the soil microbial community from the phylum to the species level under the conditions of applying strain JHY1 alone and the combined application of DA and strain JHY1. Figure 8B shows the LDA score bar chart, highlighting significantly enriched microbial taxa in different treatment groups. The findings indicated that, compared with the no-DA treatment, DA application led to notable differences in biomarker microorganisms, such as p_*Planctomycetota*, p_*Actinobacteriota*, o_*Chitinophagales*, c_*Acidimicrobiia*, and c_*Planctomycetes*. An RDA analysis was conducted to explore the correlations between environmental factors, such as NH_4_^+^-N, NO_3_^−^-N, soil ALP, soil urease, and the rhizosphere microbial abundance in the rhizosphere soil of rice under salt stress conditions (Figure 9). The results indicated that NH_4_^+^-N, soil ALP, and soil urease were positively correlated with particular microbial abundance, such as unclassified_Bacteria and *unclassified_Chloroflexi*. In addition, NO_3_^−^-N was positively correlated with other microbial abundance, such as *unclassified_Gemmatimonadaceae* and *unclassified_Alphaproteobacteria*. The correlation network among rhizosphere bacteria (Figure 10) indicated that in the soil, there was a positive correlation between the relative abundance of *Humibacillus* (Firmicutes) and *unclassified_Microtrichales* (Actinobacteria), between *Pseudolabrys* (Proteobacteria) and *Mycobacterium* (Actinobacteria), and between *Pedomicrobium* (Proteobacteria) and *Microvirga* (Proteobacteria). Meanwhile, the relative abundance of *unclassified_Saprosspiraceae* (Proteobacteria) and *unclassified_Reyranellaceae* (Proteobacteria) was negatively correlated; the relative abundance of *Nocardioides* (Actinobacteria) and *unclassified_Sandaracinaceae* (Proteobacteria) was also negatively correlated; and both *Pedomicrobium* (Proteobacteria) and *Microvirga* (Proteobacteria) showed a negative correlation with the relative abundance of *Luteimonas* (Proteobacteria).

### 3.8. PLS-PM Analysis of Plant and Rhizosphere Soil Responses to Salt Stress and DA-JHY1 Treatment

**Figure 11:**
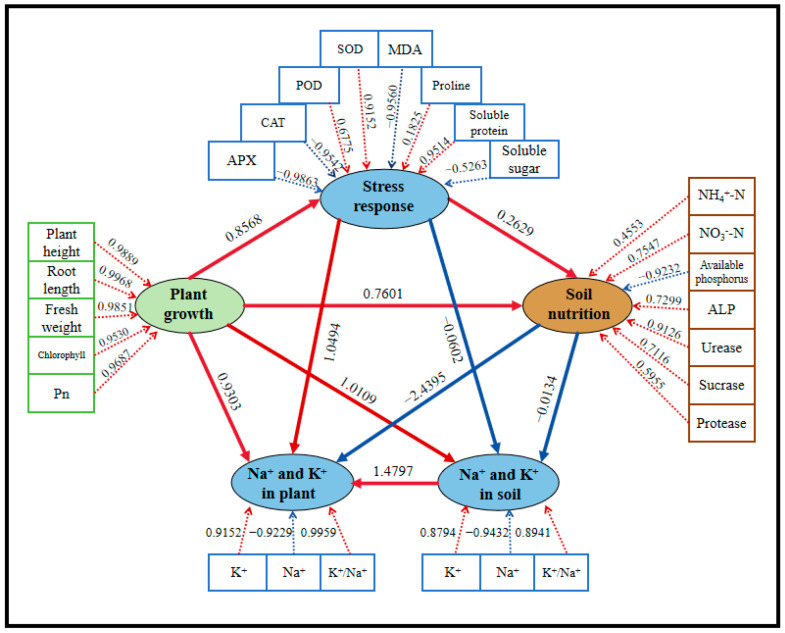
PLS-PM analysis of this study for five treatment groups. Five potential variables were included in this analysis: plant growth (comprising shoot height, root length, fresh weight, chlorophyll, and Pn), stress response (including SOD, POD, CAT, APX, MDA, soluble protein, soluble sugar, and proline), soil nutrition (composed of available phosphorus, NH_4_^+^-N, NO_3_^−^-N, available phosphorus, urease, sucrase, protease, and ALP), Na^+^-K^+^ balance in plants (including Na^+^, K^+^, and K^+^/Na^+^ ratio in plants), and Na^+^-K^+^ balance in soil (including Na^+^, K^+^, and K^+^/Na^+^ ratio in soil). Path coefficients illustrated the relationships between latent variables, with arrows indicating positive (red) or negative (blue) effects.

In PLS-PM analysis, some indicators were grouped into “potential variables”, which were abstract concepts that cannot be directly measured or observed. To elucidate the contributions of measurable indicators to latent variables, five potential variables were defined and analyzed: (1) plant growth (including shoot height, root length, fresh weight, chlorophyll content, and Pn), (2) stress response (including SOD, POD, CAT, APX, MDA, soluble protein, soluble sugar, and proline), (3) soil nutrition (including NH_4_^+^-N, NO_3_^−^-N, available phosphorus, urease, sucrase, protease, and ALP), (4) Na^+^-K^+^ balance in plants (including Na^+^, K^+^, and K^+^/Na^+^ ratio in plants), and (5) Na^+^-K^+^ balance in soil (including Na^+^, K^+^, and K^+^/Na^+^ ratio in soil). As shown in Figure 11, plant growth was significantly and positively influenced by stress response (path coefficient: 0.8568) and soil nutrition (path coefficient: 0.7601). The Na^+^-K^+^ balance in plants (path coefficient: 0.9303) and in soil (path coefficient: 1.0109) had positive impacts on plant growth, indicating that the accumulation of Na^+^ under high salt stress inhibits plant growth, whereas an increase in K^+^ and the K^+^/Na^+^ ratio is beneficial for plant growth. Antioxidant enzymes (particularly SOD and POD) and osmoregulatory substances (especially proline and soluble proteins) significantly and positively regulated plant growth (0.8568), highlighting the critical role of plant self-regulation mechanisms in mitigating stress.

## 4. Discussion

### 4.1. Effect of DA on Growth Promotion Characteristics of Strain JHY1 Under Salt Stress

Under salt stress, bacterial growth is typically inhibited, which suppresses the plant-growth-promoting effects of PGPR [51]. However, in this study, a PGPR strain capable of thriving under high salt concentrations while exhibiting robust plant-growth-promoting properties was identified. Surprisingly, under various salt stress conditions, the strain JHY1 demonstrated enhanced abilities to produce IAA, ammonia, EPSs, and to solubilize phosphorus, in addition to its intrinsic nitrogen fixation ability, showcasing its strong potential for promoting plant growth. Consistent with the previously identified wheat-promoting bacterium *Priestia aryabhattai BPR-9*, strain JHY1 was also capable of being stimulated by a specific concentration of high-salt signals to secrete higher amounts of IAA, ACC deaminase, and soluble phosphate, which were known for plant-growth-promoting properties [52]. Additionally, previous research found that EPS secretion could enhance plant ion transport capacity and stabilize the cell wall, thereby lowering ionic osmotic pressure and minimizing cellular damage [53]. This study suggested that the presence of EPSs was beneficial for the survival of both plants and rhizosphere bacteria under salt stress. Another study found that EPSs were positively correlated with the drought tolerance of the leguminous plant *R. leguminosarum* [54], further highlighting the critical role of EPSs in helping rhizosphere bacteria cope with environmental stress. In this study, it was found that the strain JHY1 was capable of growing under three different salt concentrations (150, 225, and 300 mM), with an increase in EPS secretion observed as the salt concentration rose. Previously, studies have shown that EPSs could form a protective layer on the cell surface, where the glucuronic acid group binds to Na^+^, thereby reducing the direct damage to the cells [55].

Our study also revealed that under salt stress, some critical plant-growth-promoting traits, including siderophore, were reduced. This decline could negatively impact the ability of PGPR to promote plant growth under salt stress. Interestingly, adding DA to the medium partially alleviated this adverse effect (Figure 2C–H). A previous study on the effects of DA in soybean seedlings demonstrated that DA could inhibit IAA oxidation both in vitro and in vivo by suppressing IAA oxidase [56]. Consistently, this study found that DA could enhance the IAA production of strain JHY1. The underlying reason might be that after the application of DA, DA could potentially inhibit the activity of IAA oxidase, thereby reducing the oxidation of IAA and subsequently influencing plant growth and development. In this study, although DA did not further increase EPS levels, it maintained relatively stable EPS production under varying salt stress conditions.

Regarding the mechanism of action of DA on strain JHY1, we could explore the following aspects. In plants, DA-activated antioxidant enzymes like SOD scavenge ROS within cells, thereby protecting cell membranes and proteins from damage [14,57]. Similarly, DA might exert a protective effect on bacteria under salt stress through a comparable mechanism. Additionally, as a reducing agent, DA could directly scavenge intracellular ROS [14], thereby protecting the bacterial metabolic system and maintaining the metabolic activities associated with plant growth promotion. Furthermore, previous studies have demonstrated that DA could act as a biostimulant, selectively stimulating the growth of specific beneficial microbial groups [58]. Similarly, DA might activate bacterial growth signals in this study, promoting the growth of salt-stressed microorganisms such as strain JHY1. Notably, it has also been reported that DA functioned as an exogenous signaling molecule that influenced various bacterial metabolic pathways, including the regulation of amino acid metabolism, fatty acid biosynthesis, nucleotide sugar metabolism, and pyruvate metabolism (i.e., energy metabolism) [59]. In our study, DA might affect these metabolic processes in strain JHY1, thereby influencing its PGP traits.

### 4.2. The Combined Application of DA and Strain JHY1 Has Synergistic Promoting Effect on Rice Growth Under Salt Stress

Under 225 mM NaCl stress, rice plants exhibited visible damage, including curled and withered leaf tips. All measured growth parameters, along with chlorophyll content and photosynthetic rate, showed significant reductions (Figure 3). The reason for this was that excessive salinity increases the osmotic pressure of the soil, leading to a decrease in the water potential at the root surface of the plant. As a result, this limited the uptake of water by the roots, eventually causing an insufficient water supply and osmotic stress [60]. Moreover, high NaCl concentrations led to the accumulation of Na^+^ and Cl^−^ in the plant, both of which had toxic effects on cells and interfered with the normal function of K^+^, ultimately affecting plant metabolic processes [61,62]. Additionally, high salt levels inhibited the plant’s response to N, P, Ca^2+^, Mg^2+^, and other key nutrients, resulting in an imbalance in nutrient uptake [63]. Furthermore, salt stress also triggered an oxidative stress response in the plant, which increased the production of ROS, leading to DNA damage, protein oxidation, and lipid peroxidation, which could disrupt the normal functioning of cells [64]. Regarding photosynthesis, salt stress induced stomatal closure, reduced intercellular carbon dioxide concentration, and the content of photosynthetic pigments. Additionally, salt stress damaged the membrane system, inhibited the activity of key enzymes, and blocked electron transfer from photosystem II to photosystem I in plants [65,66]. These effects ultimately led to a significant inhibition of plant growth.

Previous studies have shown that various PGPR, such as *Pseudomonas atacamensis*, *Bacillus pumilus JIZ13*, and *Enterobacter asburiae D2*, could effectively enhance rice growth and alleviate salt stress [67,68,69]. This study found that the application of either DA or strain JHY1 alone under 225 mM NaCl stress led to a certain degree of recovery in rice growth and photosynthetic performance compared to the salt stress condition. The strain JHY1 exhibited strong salt tolerance and the ability to produce IAA, ACC deaminase, solubilize phosphorus, produce ammonia, and fix nitrogen, which were key factors contributing to its effective plant-growth-promoting properties. Recent research indicated that under external stress conditions, the activity of tyrosine decarboxylase, a key enzyme in DA biosynthesis, was upregulated, leading to a concurrent increase in endogenous DA levels in plants [70,71]. This suggested that DA played a crucial role in enhancing plant stress tolerance under abiotic stress conditions by modulating physiological and biochemical responses. For example, during drought stress, apple plants overexpressing the *MdTyDC* gene (encoding tyrosine decarboxylase) exhibited higher DA levels, better growth characteristics, and enhanced photosynthetic capacity. Similarly, DA under drought stress could downregulate the expression of senescence-associated and chlorophyll degradation-related genes such as *PAO*, thereby significantly increasing chlorophyll content and delaying leaf senescence [72]. In this study, DA treatment under salt stress significantly improved growth characteristics and chlorophyll content, likely through mechanisms similar to those observed under drought stress. DA also modulated plant antioxidant enzyme levels [73]. Previous research has shown that DA possessed strong antioxidant properties, with an antioxidant capacity exceeding that of glutathione and comparable to ascorbic acid [74]. Its robust antioxidant properties stabilized photosynthetic membranes and related enzymes, enhancing photosynthetic rate and maintaining energy supply.

Combining these PGPR with other growth-promoting strategies could further amplify their beneficial effects. Our findings demonstrated that the combined application of DA and strain JHY1 yielded superior effects compared to the application of DA or strain JHY1 alone in restoring rice growth and photosynthetic performance under salt stress, with the results nearly reaching levels observed in non-stress conditions. This indicated that PGPR and DA had a synergistic effect in promoting rice growth and enhancing the salt tolerance of rice. On one hand, either DA or PGPR could promote rice growth. On the other hand, and more notably, the application of DA enhanced the growth-promoting traits of PGPR. In this study, DA increased the production of IAA, ACC deaminase, siderophores, and solubilized phosphorus, as well as nitrogen fixation by the strain JHY1, thereby further promoting rice growth.

### 4.3. DA and Strain JHY1 Coordinate Salt Resistance Through Antioxidant Enzymes, Osmotic Substances, Lipid Peroxidation, and Ion Homeostasis

Previous studies demonstrated that both PGPR inoculation and DA application could enhance antioxidant enzyme activity and increase the concentration of osmotic substances [12,74]. The antioxidant enzymes and osmotic substances in plants enhanced salt tolerance under salt stress. Antioxidant enzymes, such as SOD, POD, CAT, and APX, mitigated ROS levels, while osmolytes, including proline, soluble proteins, and soluble sugars, regulated osmotic balance both inside and outside the cells. DA not only directly scavenged ROS but also influenced the expression of antioxidant enzyme-related genes, such as MdcAPX [72]. In this study, the activities of SOD and POD were significantly reduced under salt stress compared to the control group. However, the application of either DA or PGPR alone led to an increase in the activities of these two enzymes, with PGPR exhibiting a more pronounced effect on enhancing SOD and POD activities than DA. Unexpectedly, the combined application of DA and strain JHY1 also elevated SOD and POD activities, but to a lesser extent compared to their individual applications. Additionally, CAT and APX levels showed no significant increase, and the combined treatment resulted in antioxidant enzyme levels comparable to those in the control group. The measurement results of MDA levels demonstrated that salt-stress-induced oxidative stress significantly increased lipid peroxidation in rice cell membranes, thereby compromising membrane integrity. Notably, both individual applications of DA and strain JHY1, as well as their combined treatment, effectively alleviated the extent of membrane lipid peroxidation in rice cells. This phenomenon might be attributed to the combined treatment, which could reduce oxidative stress to a low level, allowing ROS to be rapidly scavenged. As a result, the plants no longer required high levels of antioxidant enzyme activity to counteract oxidative stress. In terms of the osmotic regulatory substances, previous studies demonstrated that DA significantly increased soluble sugar content in apples and proline levels in watermelon seedlings [75,76]. This study further revealed that DA could effectively regulate the content of soluble proteins. Notably, among the three osmotic regulatory substances, the combined treatment significantly enhanced proline levels, indicating that proline played a critical role in maintaining osmotic balance between the intracellular and extracellular environments.

In this study, the CK group exhibited higher K^+^ levels than the salt-stressed treatments. This difference could be attributed to the salt-induced K^+^ efflux from the cytosol to the apoplast under NaCl stress, which led to rapid membrane depolarization and the activation of voltage-gated GORK channels, ultimately disrupting the cytosolic K^+^/Na^+^ homeostasis [77]. It has been reported that DA could upregulate the expression of *SOS1*, promoting Na^+^ efflux and enhancing NHX1-mediated Na+ transport into vacuoles. Moreover, DA could restrict the transport of Na^+^ to the shoots via *HKT1*, and *HKT1* worked in concert with the SOS signaling pathway to indirectly maintain K^+^/Na^+^ homeostasis [78,79]. Similarly, in this study, DA was found to alleviate salt stress by modulating K^+^ and Na^+^ levels in rice plants. Previous studies demonstrated that salt stress could disrupt the functionality of crucial ion channels (e.g., SOS1 and HKT1) and transporters (e.g., NHX and AKT1) in plant cells, leading to impaired Na^+^ efflux and reduced K^+^ uptake efficiency. This disruption ultimately resulted in an ionic imbalance characterized by excessive intracellular Na^+^ accumulation and the concomitant loss of K^+^ [80,81]. These findings were consistent with the observed changes in Na^+^ and K^+^ levels in rice under salt stress treatment compared to the control group in this study. Moreover, on one hand, under the protection of DA, the normal function of ion channels and transport proteins in rice was restored. ACC deaminase reduced ethylene synthesis by degrading ACC, thereby lowering ethylene levels and maintaining ionic homeostasis within cells. This effect was primarily attributed to increased IAA levels and decreased ethylene levels, which helped regulate the activity of ion channels and transport proteins in plant cells. On the other hand, IAA regulated plasma membrane potential and intracellular signaling pathways, thereby modulating ion levels. A study on tobacco demonstrated that IAA promoted the uptake and accumulation of K^+^ [82]. Consequently, this influenced the expression of sodium/hydrogen transporters, such as HKT genes, and impacted the absorption and transport of K^+^ and Na^+^ [83,84]. In this study, it was observed that under salt stress, inoculation with the IAA and ACC deaminase-producing strain JHY1 effectively maintained ionic homeostasis in rice seedlings. DA enhanced the ability of strain JHY1 to produce IAA and ACC deaminase, thereby significantly increasing K^+^ uptake, reducing Na^+^ uptake and accumulation, and improving the K^+^/Na^+^ ratio.

In addition, it was found that the application of DA/strain JHY1 alone or in combination with DA and strain JHY1 in the soil could reduce the bioavailability of Na^+^. Studies have revealed that PGPR could secrete EPSs and form biofilms, which interact with soil particles to create stable colloidal structures. The negatively charged functional groups of EPSs, such as carboxyl groups, were capable of binding harmful ions like Na^+^, thereby limiting Na^+^ uptake by plant roots and reducing bioavailable salt levels in the rhizosphere [85]. For instance, *Rhizobium* spp. producing EPSs have been shown to bind Na^+^ and significantly reduce Na^+^ accumulation in plant tissues, thereby helping to maintain K^+^/Na^+^ balance [86]. Together, these effects synergistically regulated ionic homeostasis, minimized ion toxicity, and enabled rice to exhibit optimal salt tolerance. Furthermore, the combination of DA and strain JHY1 significantly enhanced plant tolerance to salt stress through the synergistic effects of antioxidant enzymes, osmotic substances, lipid peroxidation reduction, and ion balance maintenance. This finding provided important theoretical support and practical guidance for developing new strategies to improve salt tolerance.

### 4.4. The Combined Application of DA and Strain JHY1 Improved Soil Fertility and Regulated Microbial Community Structure

NH_4_^+^-N, NO_3_^−^-N, and available phosphorus in the soil were essential for plant growth. Ammonium nitrogen was the preferred nitrogen form for rice, as it could be directly absorbed and utilized by rice roots in large quantities. In contrast, NO_3_^−^-N demonstrated a relatively lower contribution to rice growth and development [87]. Phosphorus, a crucial element for rice growth and development, played a key role in physiological processes such as cell division, energy metabolism, and protein synthesis. Available phosphorus, in particular, was readily absorbed by rice roots [88]. In this study, we found that the application of DA increased nitrogen and phosphorus levels in the soil. Furthermore, inoculation with PGPR strain JHY1, which exhibited effective nitrogen-fixing capabilities, more significantly restored soil nitrogen and phosphorus levels (Figure 5A–C). This restoration might be attributed to the ability of PGPR to secrete metabolic products, such as organic acids and phosphatases, which enhanced the levels of available phosphorus in the rhizosphere soil [89]. Additionally, PGPR directly increased rhizosphere nitrogen levels through nitrogen fixation. By altering soil microbial community structure and promoting organic matter decomposition, PGPR could also generate additional nitrogen and available phosphorus [90]. Furthermore, the growth-promoting effects of PGPR accelerate plant uptake of nitrogen and available phosphorus, thereby improving the efficiency of soil nutrient utilization and transformation.

Soil enzyme activities, closely related to plant growth, played a crucial role in nutrient cycling, maintaining soil structure, and decomposing organic residues. The activities of these enzymes served as crucial bioindicators for assessing soil health and fertility status [91]. In this study, under salt stress conditions, the activities of various soil enzymes in the plant rhizosphere, particularly phosphatase and urease, were inhibited. However, the application of PGPR had a positive influence on the activities of urease and sucrase under salt stress. The application of PGPR could regulate rhizosphere pH to a level suitable for plant growth, increase rhizosphere organic carbon, and enhance root metabolism, thereby positively influencing soil enzyme activity under salt stress [92]. Previous studies demonstrated that the application of DA effectively enhanced the activities of urease, protease, and phosphatase in the rhizosphere soil of apple replant disease, which was consistent with the results of soil enzyme activity in rice under salt stress observed in this study [24,93]. Additionally, a significant increase in soil sucrase content was also observed, which was associated with the ability of DA to regulate carbohydrate metabolism [94]. The enhancement of these rhizosphere soil enzyme activities might be attributed to the fact that DA promoted the growth of specific beneficial microorganisms, which in turn produced higher levels of these enzymes. In this study, the combined application of DA and strain JHY1 exhibited better effects on restoring soil ammonium nitrogen and phosphorus levels, as well as on enhancing soil enzyme activities. These results suggest that DA might regulate microbial communities, improve nutrient utilization, and enhance soil enzyme activity, thereby effectively alleviating the adverse effects of salt stress on plants.

DA, as a bioactive compound, not only influenced the colonization and growth of specific bacteria in the animal gut [95] but also had a direct impact on rhizosphere microbial communities in plant–microbe systems. For example, it was found that the application of DA could improve the diversity of rhizosphere microflora in apple soil under drought stress [96]. In this study, the combined application of DA and strain JHY1 under salt stress was found to increase soil microbial richness and diversity compared to the application of strain JHY1 alone. Notably, at the phylum level, DA application led to a significant increase in the relative abundance of Firmicutes, Actinobacteria, and Bacteroidetes (Figure 7E). Previous studies have confirmed that all three phyla were capable of producing IAA [97]. Some members of the phylum Firmicutes, such as the genus Bacillus, were found to enhance biofilm formation under high-salt conditions, thereby alleviating salt stress. Additionally, *Bacillus subtilis* could alleviate the impact of salt stress on plants by regulating plant hormone levels and antioxidant enzyme activities [98].

Similarly, as an important part of PGPR, Actinobacteria possessed PGP traits, including the production of plant hormones, biological nitrogen fixation, and iron-producing carriers. Over the past two decades, Actinobacteria have been increasingly recognized for their plant-growth-promoting capabilities [99]. In the root exudates of *Brachypodium distachyon*, DA levels were positively correlated with the relative abundance of two Actinobacteria (*Rhodococcus OAS809* and *Marmoricola OAE513*). Furthermore, in experiments where DA was directly added to the soil, the relative abundance of Actinobacteria significantly increased. This suggested that DA might influence plant–microbe interactions by regulating the structure of the soil microbial community, particularly the abundance of Actinobacteria. This study found that under salt stress, the application of DA also significantly increased the relative abundance of Actinobacteria in the rice rhizosphere. This could be because DA, as a signaling molecule or nutrient, promoted the growth and competitive ability of Actinobacteria, allowing Actinobacteria to dominate in salt-stressed soil environments. Although Bacteroidetes were not typically classified as PGPR, their Shannon diversity index was significantly increased in high-salinity soils, reflecting their adaptability to such environments. One study found that the relative abundance of Bacteroidetes was significantly positively correlated with soil salt content, and the bacterial diversity index also increased significantly under high-salt conditions [100]. This indicated that Bacteroides have strong adaptability and potential to restore saline–alkali land in high-salt environments. In this study, at the genus level, the relative abundance of *Chryseolinea* significantly increased in the DA-treated group. A previously identified strain of *Chryseolinea* demonstrated certain tolerance in the soil, being able to grow under low-to-moderate salinity and pH conditions [101]. The interactions among soil microbial communities, nutrients, and enzyme activities collectively contributed to soil homeostasis, which was crucial for plant growth. In our study, RDA revealed that the microbial communities in DA-treated groups were positively correlated with rice-growth-promoting environmental factors, including ammonium nitrogen, soil urease, and alkaline phosphatase levels. In conclusion, the application of DA favored the increase in salt-tolerant bacteria within Firmicutes, Actinobacteria, and Bacteroidetes, thereby regulating the microbial community structure under salt stress. This, in turn, enhanced soil nutrient availability and enzyme activity, ultimately contributing to the increased salt tolerance of rice.

The increased abundance of Actinobacteria and Firmicutes in salt-stressed soils could contribute to the stability of the ecosystem, particularly in maintaining the decomposition of organic matter, nutrient transformation, and suppression of pathogens [102,103,104]. However, such shifts might also lead to imbalances in the microbial community structure, potentially suppressing other important phyla, such as Proteobacteria, which play critical roles in soil functioning. Therefore, in future studies, it would be beneficial to monitor the long-term dynamics of the entire soil microbial community to conduct a more comprehensive assessment of their ecological roles and interactions.

### 4.5. DA and PGPR: Safety and Future Research

The current study was primarily conducted under greenhouse conditions, with rice seedlings being transplanted into pots and cultivated for 30 d after germination. Although this study observed significant positive effects of the combined treatment strategy on rice growth and key physiological parameters during this stage, with enhanced salt tolerance in rice, real-world agricultural planting environments are highly complex and variable. Moreover, crops may exhibit different characteristics across various growth stages. Therefore, additional long-term and large-scale field trials that cover the entire growth cycle are still necessary to verify the sustained effectiveness and feasibility of this synergistic strategy under various soil types and environmental conditions.

Soil ecological safety is of vital importance. First, DA is a common catecholamine that is widely present in both plants and animals and is also an endogenous compound in plants. Current research indicated that DA had significant positive effects on plant stress resistance, with no evidence of harmful impacts on plants. Future studies could focus on determining the safe concentration ranges of DA in soil and plants. In addition, DA has many advantages in the agricultural field, such as low cost, easy production, simple structure, and high biosafety. It has been reported that DA could be sustainably produced from waste softwood lignin, with a theoretical production cost reduced to 2.2 million CNY per ton [105]. Second, strain JHY1 is the PGPR isolated from rhizosphere soil, and its re-application to soil is relatively safe. *Proteus* is a common genus of bacteria in soil. Although some strains of *Proteus* are opportunistic pathogens, they show more positive aspects in natural environments [106]. An increasing number of studies have found that *Proteus* strains could enhance plant stress resistance [106,107]. However, before applying in the field, it is necessary to conduct a comprehensive pathogenicity assessment of the strains and carry out continuous monitoring of soil ecological safety. Although the addition of exogenous compounds or microorganisms to the soil might impact the functioning of soil ecosystems, DA and PGPR are relatively safe.

This study has found that DA has a positive effect on the growth of strain JHY1 and its PGP traits. However, the underlying molecular mechanisms remain unclear. Future research can conduct molecular-level analyses of strain JHY1 using isotope-labeling techniques to investigate the metabolic pathways of DA within strain JHY1. Meanwhile, integrating multi-omics approaches, including genomics, transcriptomics, proteomics, and metabolomics, along with the detection of key physiological and biochemical indicators, will be essential. The focus should be on exploring the differential gene expression and transcriptional regulation changes in the strain under DA treatment to systematically elucidate its molecular mechanisms of action.

## 5. Conclusions

Overall, salt stress significantly hampered rice growth by inhibiting photosynthesis, increasing lipid peroxidation, affecting antioxidant enzyme activities, reducing available soil nutrients and enzyme activities, and altering the K^+^/Na^+^ ratio in rice and soil. The salt-tolerant strain JHY1 possessed various PGP traits, such as high levels of IAA production and ACC deaminase activity. In addition, exogenous DA treatment aided the growth of strain JHY1 under salt stress. Both strain JHY1 and DA treatments mitigated the inhibitory effects of salt stress on rice growth. When strain JHY1 and DA were applied in combination, the mitigating effect of salt stress was more pronounced, as evidenced by significantly enhanced photosynthesis, improved soil nutrients and enzyme activities, regulated ion balance, and the modulated rhizosphere microbial community structure. These combined effects contributed to the enhanced salt tolerance of rice under saline conditions. This study provides a theoretical basis for the synergistic application of JHY1 and exogenous DA to enhance rice growth performance under salt stress.

## Data Availability

The original contributions presented in this study are included in the article; further inquiries can be directed to the corresponding authors.
